# Differentiation and Distribution of Marrow Stem Cells in Flex-Flow Environments Demonstrate Support of the Valvular Phenotype

**DOI:** 10.1371/journal.pone.0141802

**Published:** 2015-11-04

**Authors:** Sasmita Rath, Manuel Salinas, Ana G. Villegas, Sharan Ramaswamy

**Affiliations:** Department of Biomedical Engineering, Florida International University, Miami, FL, 33174, United States of America; National Institutes of Health, UNITED STATES

## Abstract

For treatment of critical heart valve diseases, prosthetic valves perform fairly well in most adults; however, for pediatric patients, there is the added requirement that the replacement valve grows with the child, thus extremely limiting current treatment options. Tissue engineered heart valves (TEHV), such as those derived from autologous bone marrow stem cells (BMSCs), have the potential to recapitulate native valve architecture and accommodate somatic growth. However, a fundamental pre-cursor in promoting directed integration with native tissues rather than random, uncontrolled growth requires an understanding of BMSC mechanobiological responses to valve-relevant mechanical environments. Here, we report on the responses of human BMSC-seeded polymer constructs to the valve-relevant stress states of: (i) steady flow alone, (ii) cyclic flexure alone, and (iii) the combination of cyclic flexure and steady flow (flex-flow). BMSCs were seeded onto a PGA: PLLA polymer scaffold and cultured in static culture for 8 days. Subsequently, the aforementioned mechanical conditions, (groups consisting of steady flow alone—850ml/min, cyclic flexure alone—1 Hz, and flex-flow—850ml/min and 1 Hz) were applied for an additional two weeks. We found samples from the flex-flow group exhibited a valve-like distribution of cells that expressed endothelial (preference to the surfaces) and myofibroblast (preference to the intermediate region) phenotypes. We interpret that this was likely due to the presence of both appreciable fluid-induced shear stress magnitudes and oscillatory shear stresses, which were concomitantly imparted onto the samples. These results indicate that flex-flow mechanical environments support directed *in vitro* differentiation of BMSCs uniquely towards a heart valve phenotype, as evident by cellular distribution and expression of specific gene markers. *A priori* guidance of BMSC-derived, engineered tissue growth under flex-flow conditions may serve to subsequently promote controlled, engineered to native tissue integration processes *in vivo* necessary for successful long-term valve remodeling.

## Introduction

Heart valves play an important role in controlling unidirectional blood flow. However, birth defects or infections (e.g. rheumatic fever) can cause one or more of the heart valves to malfunction, which may lead to critical valve anomalies in children. Advances in implant design and surgical techniques have substantially augmented the success of prosthetic heart valves in adult patients. However, the efficacy of these implants is severely limited in pediatric patients due to their inability to promote somatic growth and valvular tissue remodeling; as a result, multiple major surgeries and re-operations are commonplace, which places a significant health burden on the growing child.

Similar to native valves, a tissue engineered heart valve (TEHV) has the ability to adapt and evolve with the living host and is conceptually considered a permanent solution for the treatment of heart valve disease [1]. During each cardiac cycle, native valves are continuously subjected to mechanical stress as a result of blood flow; for example, aortic valve leaflets experience peak fluid-induced shear stresses of approximately 5–6 dyne/cm^2^ in mid systole [2–4]. Mechanical stimuli, when applied to developing cardiovascular tissues, alter gene expression-promoting tissue remodeling events, which in turn enhances specific mechanical and phenotypic characteristics. Native heart valves are subjected to highly complex cyclic, tensile and fluid-induced stresses[4]. In an engineered heart valve tissue context, mechanical stimuli, particularly those that incorporated fluid-induced shear stress, have enhanced progenitor cell differentiation pathways and construct tissue properties for the valve application [5–8]. For example, Hoerstrup et al. performed experiments using a pulsatile flow bioreactor device that enhanced TEHV tri-leaflet structures with ∼ 300% increase in the collagen extracellular matrix (ECM) content compared to statically cultured counterparts. Elsewhere, bioreactors have been built to couple any combination of flow, cyclic stretch and cyclic flexure (FSF bioreactors), which have also verified that coupled mechanical stimuli significantly promote ECM production; in particular, the combination of steady flow with cyclic flexure [9,10] relevant to heart valves, Vermot et al. showed that blood flow-induced oscillatory shear stresses (OSS) directly modulate the normal expression of a transcription factor from the kruppel-like factor gene family, KLF2A, in a zebra fish model. The KLF2A gene is critically involved in valvulogenesis, whose absence results in defective heart valves [11].

The generation of a functional TEHV has remained elusive in part, due to lack of information regarding the mechanobiological events necessary to optimize the *in vitro* culture process. Nonetheless, under mechanical guidance, tissue engineering feasibility studies have thus far demonstrated that native valvular cells can recapitulate valve structure with adequate mechanical strength and morphology [12–16]. In addition, non-valvular cells, such as bone marrow stem cells (BMSCs), saphenous vein endothelial cells (ECs), ascending aorta myofibroblasts and umbilical cord-derived cells, have exhibited increased production of valvular ECM, DNA content and endothelization *in vitro* under mechanical conditioning states [17–22].

BMSCs in particular have shown considerable promise for heart valve tissue engineering, as they are multipotent stem cells with a minimal risk of immunogenicity, are void of ethical/legal concerns and can be obtained and culture-expanded easily; typically, BMSCs can be isolated, purified and expanded to a large number in a matter of days [23]. BMSCs maintain extensive differentiation, proliferation and clonogenic capacity *in vitro*. Human BMSCs respond to mechanical conditioning and have been shown to produce heart valve ECM components *in vitro* [24,25]. Engelmayr et al [9,26], described pioneering work on elucidating the effects of combined cyclic flexure and steady flow states (flex-flow), which served to significantly promote engineered collagen in *de novo* engineered heart valve tissues derived from BMSCs. However, a fundamental precursor to TEHV studies evaluated at the tissue scale is the need to understand the process by which valve-relevant mechanical stimuli can regulate cell fate, particularly cell differentiation in the context of stem cell sources. Thus, in this study, our primary goal was to determine unique responses of BMSCs to flex-flow conditions in the promotion of valvulogenesis.

## Materials and Methods

### BMSCs Culture and Expansion

BMSCs isolated from human bone marrow, characterized and tested with stromal, stem, and hematopoietic marker, were purchased in frozen vials (Fisher Scientific, Pittsburgh, PA). Cells were recovered from dry ice packaging, and were immediately thawed and diluted with a pre-warmed culture medium to reduce the toxic effects of cryopreserve reagents upon arrival. Approximately 5 x 10^5^ cells were transferred to a T75 vented cell culture flask and placed in an incubator supplied with 5% CO_2_ at 37°C with 95% humidity. Flasks were fully confluent at 1 week. An average of 2 x 10^6^ cells were obtained at each passage. A freshly prepared stem cell culture medium with a 10% advanced stem cell undifferentiated growth supplement, 1% penicillin and streptomycin (Thermo Scientific^™^ HyClone^™^, Fisher Scientific) medium was used for cell culture and expansion. An aseptic cell and tissue culture environment was dedicated for the entire duration of the experiment. BMSCs at passages 6 to 8 were utilized for subsequent tissue engineering experiments.

### Scaffold Preparation and Cell Seeding

**Fig 1:**
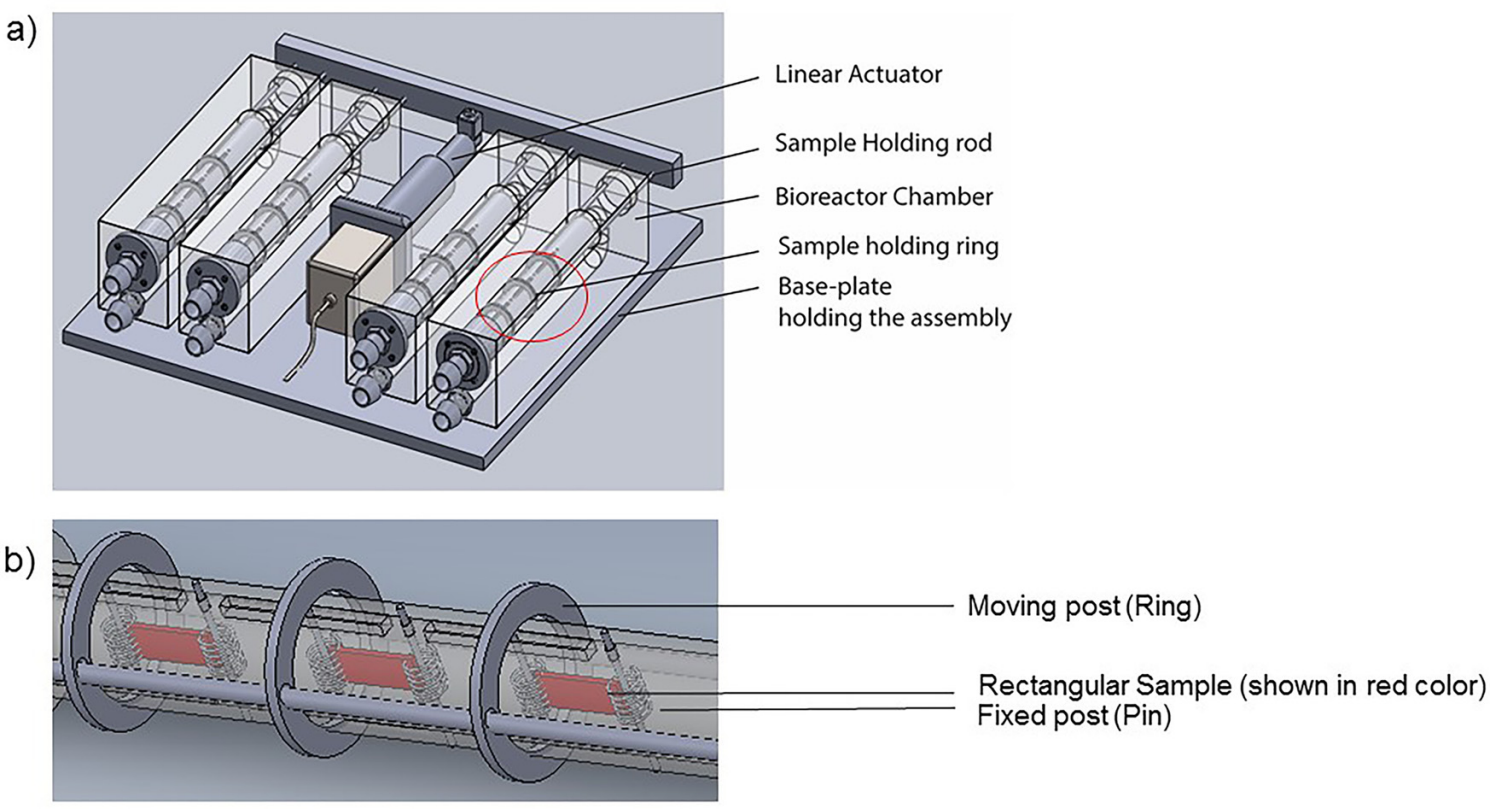
a) Schematic diagram of the custom built U-shaped bioreactor connected to a linear actuator which guides the rods that threads through samples, permitting them to bend and straighten. b) Inset: shows three samples inside the conditioning chamber that can be moved one end (ring) and is fixed on the other (pin). In the current study, the actuator was set to a 1 Hz frequency to permit cyclic flexure while the pump operated at a steady flow rate of 850 ml/min.

An equal ratio of poly-glycolic acid (PGA) and poly-L-lactic acid (PLLA), nonwoven polymer felt was utilized as the scaffold material (Biofelt, Biomedical Structures, Warwick, RI) [9,27,28]. Specimens measuring 17 mm long, 6 mm wide and 1 mm thick (n = 12) were cut and two metallic springs were carefully attached to either end of the scaffold. This assembly was required for dynamic tissue culture in our customized U-shaped bioreactor device (Fig 1); additional drawings and details can be found in Ramaswamy et al. [29]. Prior to cell seeding, scaffolds were gas sterilized with ethylene oxide (EtO; AN 306, Anprolene, Andersen Products Inc, HawRiver, NC) for 12 hours and treated with 70% ethanol. Aeration procedures were performed, as per the recommendations described in the manufacturer’s instruction guide [30]. The following protocol was used to complete the seeding procedure: Approximately 90% confluent flasks were rinsed with Dulbecco’s Phosphate-Buffered Saline (DPBS, Fisher Scientific) buffer. Next, 0.25% trypsin and ethylenediaminetetraacetic acid (EDTA) solution were added and incubated at 37°C for 3 minutes. An equal volume of serum was used to neutralize the trypsin solution. Cell suspensions were collected in15 ml conical tubes and centrifuged at 1700 rpm for 5 minutes. A cell pellet was retrieved by removing the supernatant and was suspended with freshly prepared tissue culture media for further usage. A single scaffold was seeded with 2 x 10^6^ BMSCs and suspended in 20 ml of tissue culture media in 50 ml vented conical tubes (Product # TP87050, TPP, TubeSpin Bioreactor, Zollstrasse 7, CH-8219 Trasadingen, Switzerland). The tissue culture media was comprised of Dulbecco’s modified Eagle’s medium (DMEM, Fisher Scientific), supplemented with 10% fetal bovine serum (Atlanta Biologics, GA, USA), 1% penicillin and streptomycin (Thermo Scientific^™^ HyClone^™^; Fisher Scientific), 2ng/ml basic fibroblast growth factor (bFGF, Corning^™^; Fisher Scientific) and 82 μg/ml ascorbic acid 2 phosphate (AA2P, Sigma-Aldrich). Subsequently, these tubes were placed in a rotisserie (Labquake^™^ Rotisserie Hybridization Rotators, Thermo scientific, USA) at 8 RPM inside a cell and tissue culture incubator. Media was changed every two days and the BMSC-seeded scaffolds were cultured under rotisserie culture for a total timeframe of 8 days.

### Tissue Engineering Experiments

Beyond the first 8 days of rotisserie culture, samples were randomly assigned to one of four different treatment groups and underwent an additional period of tissue culture for 14 days. These groups were (n = 12 specimens/group): 1) No flow (Static Controls), 2) Steady flow-alone (flow), 3) Specimen cyclic Flexure-alone (flex) and 4) Combination of steady flow and specimen cyclic flexure (flex-flow). For the mechanical conditioned groups, a custom-built U-shape bioreactor, which was previously described extensively, was used [29]. This device is connected to a peristaltic pump and an environmentally sealed linear actuator for experimental purposes. The experimental set-up consisted of four identical conditioning chambers, with each chamber containing 3 samples. Specimens were fixed with a pin at one end while the other end was attached to a circular moving post. For Flex and Flex-Flow experiments, the moving post was moved linearly in the axial direction to initiate specimen cyclic flexure using the linear actuator (frequency of 1 Hz). For the Flow and Flex-Flow cases, a peristaltic pump (Master flex L/S, model # 7523–80, Cole Parmer, model # 7535–04, East Bunker Court Vernon Hills, IL, USA) was used to maintain a continuous flow rate of 850 ml/min (S1 File).

### Collagen Content

Specimens from all four groups were removed following 22 days (n = 3 per group) and subjected to collagen biochemical assays (Biocolor life science assays, Carrickfergus, County Antrim, UK). Digested collagen samples were quantified similar to methods described previously [28]. Samples were digested with a solution of 0.5 M acetic acid (Sigma) and pepsin (1 mg/ml Pepsin (P7000), Sigma). Digestions were carried out for 16 hrs on a rocker (Orbitron Rotator; Boekel Scientific, Feasterville, PA) at 4°C. Collagen extracts were then assayed according to the *in vitro* tissue procedure provided by Sircol soluble collagen assay kit (Biocolor Ltd.). A multi-mode microplate reader was (Synergy HT, Biotek instrument, Inc, # 7091000) was set to an absorbance of 555 nm to obtain the collagen concentration.

### Histology

In order to detect the presence of key valvular ECM components (collagen, glycosaminoglycans (GAGs) and elastin) the cultured engineered tissues and freshly harvested porcine native aortic valve leaflets (Mary’s Ranch, Miami, FL; as a positive control) were subjected to histological processing. At room temperature, the samples were washed with DPBS and fixed with 10% formalin overnight, with a 20:1 volume fraction. After fixation, 2 mm by 2 mm tissue sections were cut with the help of a pair of scissors and embedded in the tissue freezing medium (Catalog #:19636–4, Polysciences, Warrington, PA). To retain structure, tissues were incubated with the liquid freezing medium at room temperature in a petri dish for 2 hrs. Next, the liquid freezing medium was also separately added to plastic molds (Fisher Scientific) and frozen in liquid nitrogen temperature. A white solid bed of the freezing medium was obtained after ~ 1 minute. At this point, the prepared tissue section was carefully placed on the frozen solid bed inside the mold and more freezing medium was added to immerse the sample. The mold was subsequently snap-frozen in liquid nitrogen. Finally, serial sections (12 μm thickness) parallel to the tissue surface were cut using a cryostat microtome (Leica CM3050 S, Buffalo Grove, IL, United States). The sections were mounted onto glass slides (True bond 380, Newcomer supply, Middleton, WI). Histological stains were applied onto the tissue sections using the supplied dyes from the manufacturer (Russell-Movat Pentachrome staining kit, American MasterTech, Lodi, CA) for subsequent microscopic visualization.

### Immunofluorescence staining

Immunofluorescence staining of CD31 and α-SMA cell surface proteins was performed (n = 3 specimens/group) after 22 days for evidence of endothelial and myofibroblastic phenotypes, respectively. This study focused on two valve relevant proteins, such as cluster of differentiation 31 (CD 31) and alpha-smooth muscle actin (α-SMA). CD31 is known to be expressed on the surface of EC and α-SMA in SMC, as well as myofibroblast-like cells in valve interstitial cells. Each sample was sectioned along the tissue and three different layers, i.e. the top surface layer, the deep middle core layer and the bottom surface layer, were immunostained with anti α-SMA and anti CD31, and were evaluated for SMC and EC expression. A porcine aortic heart valve isolated from a heart (Mary’s Ranch Slaughterhouse, Miami, FL) was dissected and assigned as positive control. The valve leaflets were immunostained for EC and SMC expression following the same protocol.

Sample fixation, embedding and sectioning procedures were identical to the histological protocol described earlier (“Histology” sub-section in the “Materials and Methods”). Next, for immunofluorescence detection, the following staining procedure was followed: tissue sections mounted on the glass slides were treated with 0.1% Triton X-100 for 3 to 5 minutes to enhance the permeability of the cell cytoplasm (this step was excluded for CD31 staining). Additional washing steps were performed three times for 5–10 minutes with DPBS. Blocking of nonspecific epitopes was facilitated by adding 1% goat serum in DPBS for 30 minutes. The primary antibodies used were mouse monoclonal anti-CD31 (ab24590, Abcam, Cambridge, MA, USA) and mouse monoclonal anti-α SMA (ab18147, abcam, Cambridge, MA, USA). An overnight incubation at 4°C was done. Samples were washed with wash buffer (DPBS+ 0.01% Triton-X 100) to reduce background. Secondary antibody (Goat polyclonal anti-mouse IgG (H+L) (DyLight 488) (Fisher Scientific) with 1% goat serum) was added and incubated overnight at 4°C for both CD31 and α-SMA immunofluorescence staining. Glass slides with stained sections were viewed under a fluorescent microscope (Olympus BX51, Center Valley, PA). For CD31, a depth of 92±10.58 μm from the top of the surface was considered as the top layer, a depth of 76±4 μm from the bottom surface was considered as the bottom layer, and a 264±18.33 μm–652±17.43 μm depth from the top surface was considered as the deep middle core layer. Similarly, for α-SMA, a depth of ~140±17.43 μm from the top surface was considered as the top layer, a depth of ~ 108±12 μm from the bottom surface was considered as the bottom layer, and a depth of 336±13.85 μm–464±21.16 μm from the top surface was considered as the deep middle core layer.

#### Image Analysis

Signal intensity values for CD31 and α-SMA staining images (sample size, n = 3) were quantified using the following protocol (ImageJ, NIH; Bethesda, MD). A rectangular region of interest (ROI) with an area of 10,000 pixels was defined. Three different ROIs (replicates, r = 3) with maximum intensity were selected and measured. Average intensity of the ROIs was recorded in arbitrary units (AU). Care was taken to avoid any false positive signals [31].

### Quantitative Real-Time Polymerase Chain Reaction (QRT-PCR)

After 22 days of tissue culture, nascent tissues from the four groups were evaluated for gene expression and quantification of a limited number of valve-related markers in order to verify the immunostaining results. Quantitative real time-polymerase chain reaction (QRT-PCR) was performed, as previously described [32–35]. Engineered tissues were washed with DPBS and total RNA was isolated according to the manufacturer’s protocol (SV Total RNA Isolation System,Promega) Preparation of Lysates from Small Tissue Samples (≤30mg). Briefly, 1ml of lysis buffer was added to 30 mg of tissue, and was flash frozen and homogenized with a homogenizer at high speed until no visible tissue fragments remained in the mixture. A volume of 175 μl of tissue lysate was transferred to a 1.5 ml micro-centrifuge tube. The pellet was shifted to a 1.5ml RNase-free micro-centrifuge tube to perform the RNA isolation. Total RNA was purified with the SV Total RNA Isolation System (Promega). Isolated mRNA concentration and quality was verified utilizing a spectrophotometer (Varian Carry 300). A 60 fold dilution was prepared for all sample measurements. RNA purification data is provided in supplemental data sheet (S3 File). 1 μg of total RNA was used for reverse transcription reaction and the cDNA was synthesized using an oligo (dT) _15_ primer provided by GoScript ^™^ Reverse transcription system (Promega). QRT-PCR was performed using GoTaq_qPCR Master Mix (Promega). Signal intensities were detected with a Step-One Real-Time PCR System (Applied Biosystems). The PCR mixture contained forward and reverse primers and SYBR green I dye reagent, along with the cDNA obtained from reverse transcription. The primers and genes were selected from the referred sources and all primers were purchased (Sigma Aldrich). Primer sequences (Table 1) GAPDH, YARS, KLF2A sequences were obtained using the BLAST program, National Center for Biotechnology Information (NCBI), FZD2 and MLC1F were obtained from Brand et al. Briefly, the conditions used for the experiment were as follows: PCR tubes (Applied Biosystems, Grand Island, NY) were held at 95°C for 2 min before the cycle started to activate the Taq polymerase. The cycling parameters were 95°C for 5 sec; 60°C for 45 sec; 95°C for 15 sec. The change in threshold cycle (ΔC_t_) values was averaged and normalized with GAPDH, an endogenous gene, using the ΔΔC_t_ method described in the reagent guide (Applied Biosystems) [36]. The expression of gene fold changes was calculated as 2^- ΔΔCt^, and the gene expression ratio of four groups (Static, Flow, Flex and Flex-Flow, PHV) were compared for further analysis. Melt curve analysis was performed after each complete QRT-PCR cycle to check any contamination, primer quality or duplicate amplification [37]. Melting temperature (T_m_) of GAPDH, YARS, FZD2, MLC1F, KLF2A were recorded as (82.6±0.001)°C, (80.05±0.007)°C, (81.97±0.08)°C, (79.91±0.09)°C, and (79.92±0.001)°C respectively. Melt curve for each group is provided in supplemental data (S4 File).

**Table 1:** Primer sequences utilized for RT-PCR analyses in this study.

Gene ID	Gene Name	F: Forward Primer (5’-3’)	Gene References
		R: Reverse Primer (5’-3’)	
GAPDH	Gglyceraldehyde-3-phosphate dehydrogenase	F: AATGAAGGGGTCATTGATGG	[35]
		R: AAGGTGAAGGTCGGAGTCAA	
YARS	Ttyrosyl-tRNA synthetase	F: CCTCCAAATTGGGCATCTAC	[38]
		R: GGAGCTGAGGTGGTAAAGCA	
FZD2	Frizzled class receptor 2	F: CGGCCCCGCAGCGCCCTGCCC	[39]
		R: ACACGAACCCAGGAGGACGCAGGCC	
MLC1F	Myosin light chain 1	F: GAGTTCTCTAAGGAACAGCAGG	[39]
		R: CTGCGTGTCTTTGACAAGGAAGGCAATGG	
KLF2A	Kruppel-like factor 2a	F: CCGTGTGCTTTCGGTAGTG	[11]
		R: AAGAGTTCGCATCTGAAGGC	

### Computational Fluid Dynamics (CFD)

In order to quantify the fluid-induced stresses imparted on the bioreactor specimens, we conducted CFD simulations. For the cases of flex-flow and cyclic flexure alone, we incorporated moving boundary analyses; specifics on the CFD approaches are described in detail in our previous work [40]. The four cases of flow-alone, cyclic flexure-alone, combined flow and flexure, and no flow were simulated. We used an inlet velocity boundary condition 0.1067 m/s for the cases of flex-flow and steady flow-alone, which represented the experimentally prescribed flow rate of 850 ml/min. No slip conditions were prescribed to the bioreactor walls, while the walls the samples were set equal to the velocity of the grid for the flex-flow and cyclic flexure-alone. The outlet of the bioreactor was set to a zero relative-pressure boundary condition. All simulations were run utilizing a Newtonian, viscous model, with laminar flow conditions, with the following fluid material properties: density = 1.01g/cm^3^ and dynamic viscosity = 1.27 cp (CFx, Ansys Inc., Canonsburg, PA). The results were analyzed after a convergence criterion of 1x10^-9^ set for each of the momentum, continuity, and mesh displacement equations was satisfied. All simulations were conducted in a Hewlett Packard work station with intel(R) Xeon(R) CPU, x5550@ 2.67GHz (2 processors), with 16.0 GB installed memory and 64-bit Windows 7 operating system.

In order to denote the coupled effects of shear stress magnitude and the temporal oscillations in the flow, we utilized an oscillatory shear index (OSI)-scaled shear stress magnitude (OSI-$\vec{\left|\tau \right|}$) which we previously defined [40] as:

$$\text{OSI}-\vec{\left|\tau \right|}\;=2*\text{OSI}*\text{TSSM}$$

Where TSSM is the time-averaged shear stress magnitude. The OSI [41] itself is defined by:

$$\text{OSI}=\;\frac{1}{2}\left(1-\frac{\text{abs}\left(\int_{0}^{\text{T}}\tau \text{dt}\right)}{\int_{0}^{\text{T}}\text{abs}\left(\tau \right)\text{dt}}\right)$$

Where, “τ” is the fluid-induced shear stress, “T” is the period and “t” is time. The OSI ranges from 0 to 0.5; an OSI of 0 represents unidirectional flow while an OSI of 0.5 signifies a high degree of temporal oscillations in the flow (S5 File).

### Statistical Analyses

One way analysis of variance was conducted to test any significant differences between the four groups investigated for collagen production, image analysis and gene expression outcomes (n = 3 samples/group/outcome). A Tukey’s post hoc test was subsequently performed to determine significant differences between groups. All statistical analyses were conducted using the statistical package for the social science (SPSS) software (V16, IBM, Armonk, NY). Significant differences between groups were observed to have occurred at a significance level of p < 0.05.

## Results

### Collagen Content

Physiologically-Relevant Flex-Flow Mechanical Conditioning Further Augments Collagen in Engineered ECM: Collagen is a key structural protein in heart valve tissues which undergo continuous remodeling under cyclic flexure, cyclic stretch and pulsatile flow mechanical states. Towards the tissue engineering of heart valves, we examined how flexure and/or steady flow modes of mechanical stimuli at physiologically relevant scales can promote collagen content in the de novo tissues derived from BMSCs.

**Fig 2:**
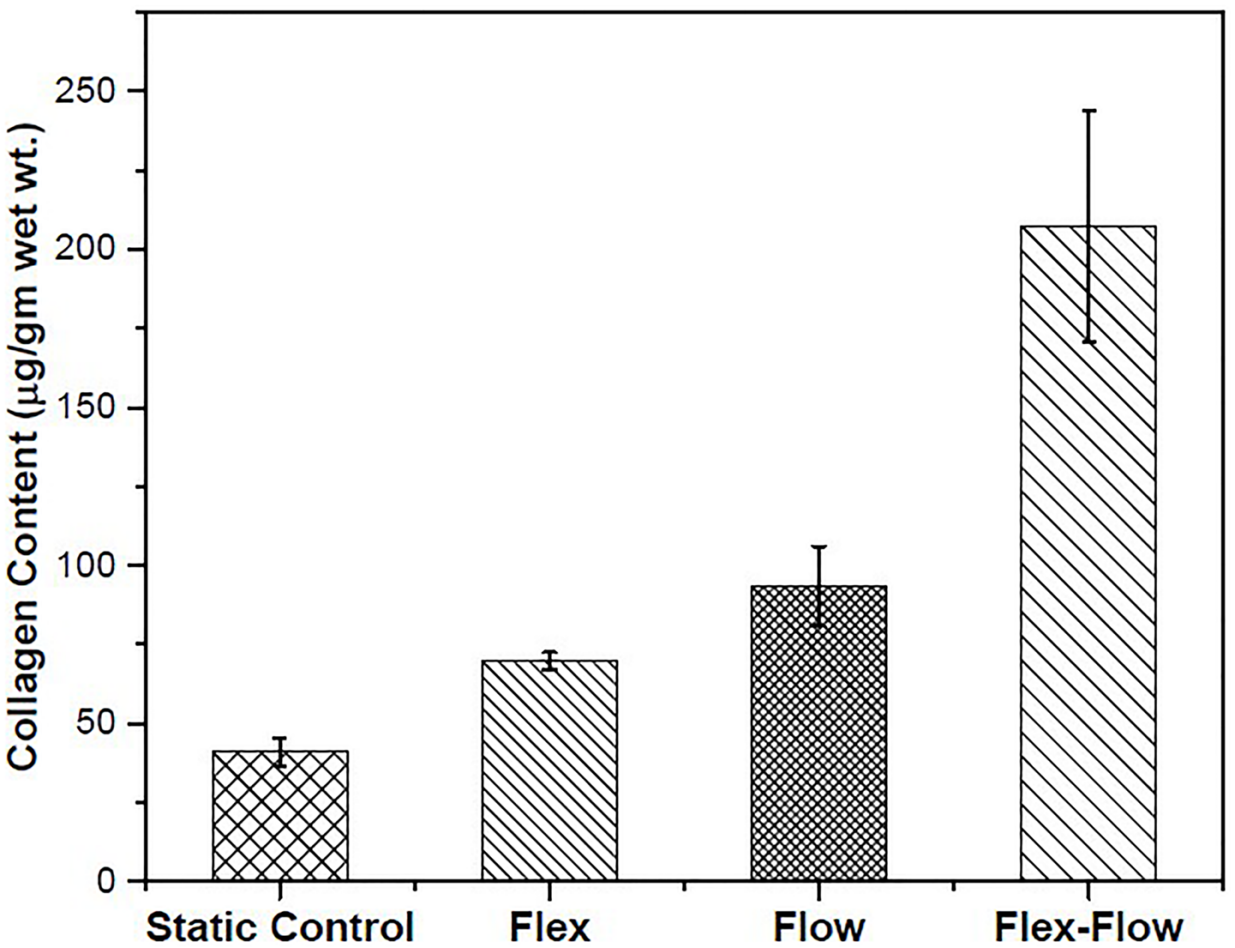
Collagen content in specimens derived from each group investigated. The Flex-Flow group produced significantly (p < 0.05) higher collagen compared to all other groups.

The average collagen production in the static group was found to be 40.91±4.23 (no flow, no flexure), 93.29±12.6 (flow-alone), 69.75±2.68 (cyclic flexure-alone), 207.38±36.61 (flex-flow) μg/g wet weight (Fig 2). Collagen content of the flex-flow group was found to be significantly higher (p<0.05), compared to the other three groups. There was no significant difference found among static, flow only and flex only groups. This observation is in agreement with previous findings [9]. For comparison, the collagen content of porcine aortic valve leaflets was evaluated (n = 3 leaflets) and found to be 2141.17±491.56 μg/g wet weight (S2 File).

### Histology

Flex-Flow conditioning of BMSC-seeded scaffolds results in increased presence of collagen and GAGs in the ECM: BMSC-derived engineered tissues exposed to valve-relevant mechanical stimuli were subjected to histological processing in order to visualize the three key ECM components of the valvular matrix: collagen, GAGs and elastin.

**Fig 3:**
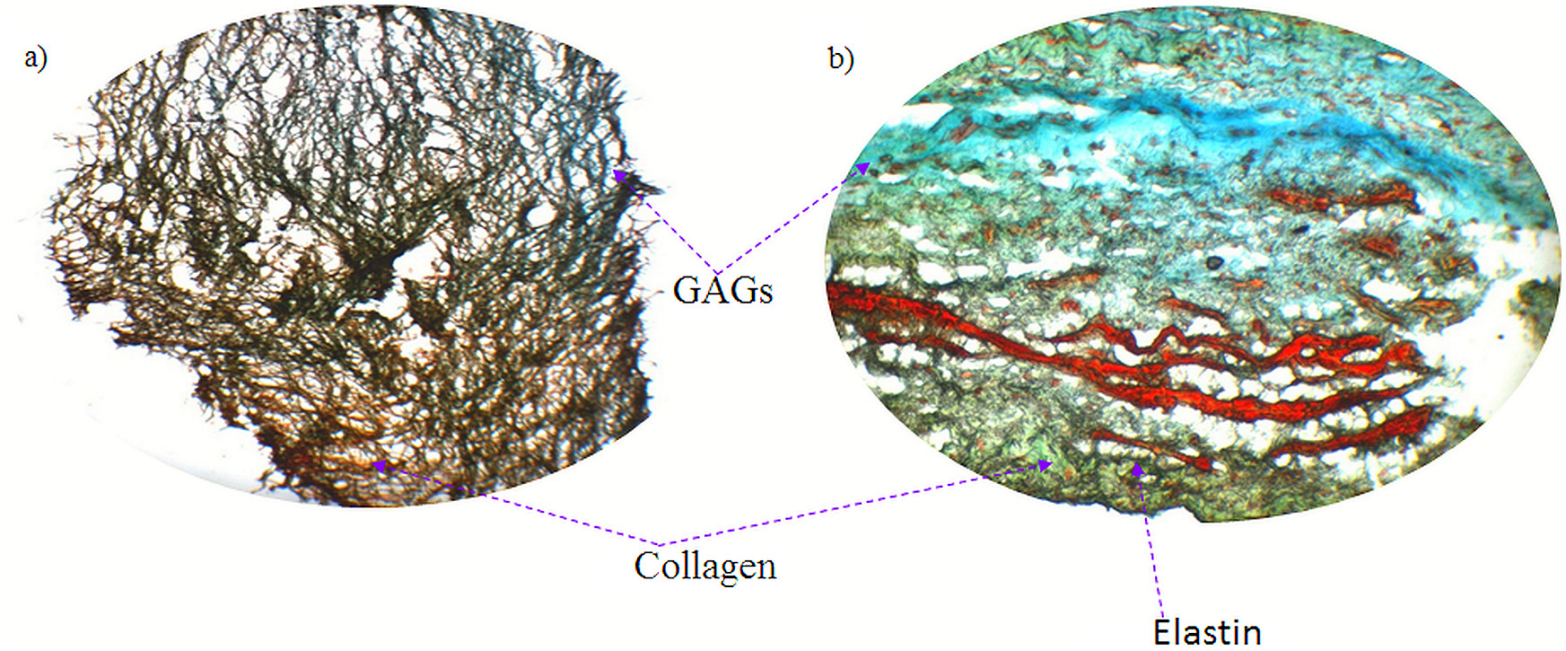
Russell’s Movat pentachrome histological staining of: a) through-thickness section of BMSC-seeded scaffold after 22 days of culture (8 days static + 14 days Flex-Flow conditioning, b) through-thickness section of native porcine aortic valve leaflet (+ve control). Color code Blue: Glycosaminoglycans (GAGs), Yellow-Green: Collagen, Black: Elastin, Red: Muscle, of the three principal components of native valve ECM, Collagen and GAGs were clearly visible within the tissue engineered construct; however the presence of Elastin was inconclusive and likely absent.

Specimens that underwent Flex-Flow mechanical conditioning for 14 days in the bioreactor following 8 days of static culture displayed evidence of collagen and GAGs within the engineered tissue ECM (Fig 3). However the presence of elastin was found to be inconclusive and likely to be absent, which is typically the case for *in vitro* grown valvular tissues [42].

### Immunofluorescence staining

Flex-Flow conditions promote valve-like distribution of BMSC-differentiated endothelial cells and smooth muscle cells in the engineered tissues: To assess how differentiated BMSCs distribute within de novo ECM after dynamic culture, immunostaining of engineered tissue serial sections from the surface to deeper layers within the constructs was performed.

**Fig 4:**
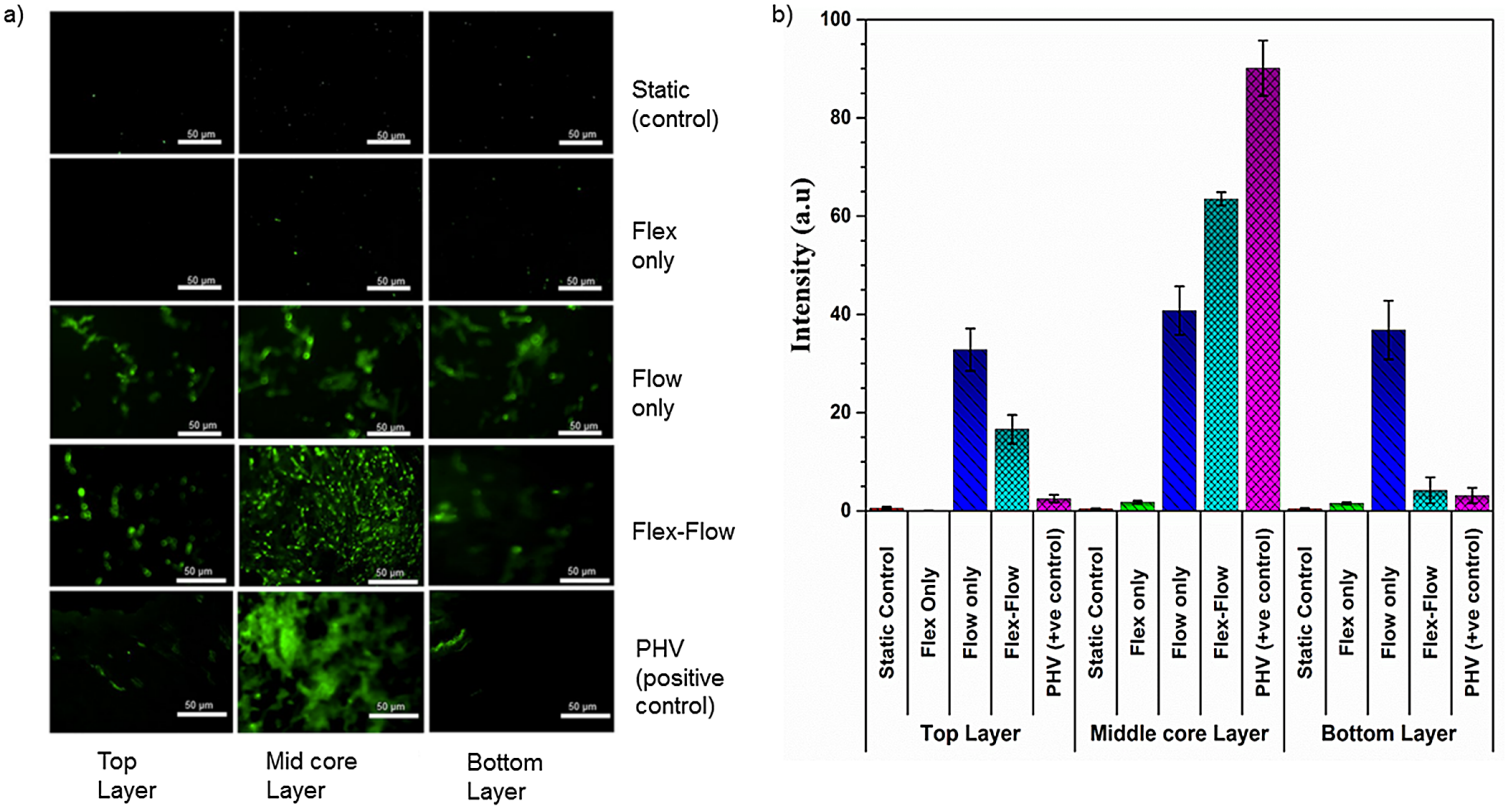
a) Immunofluorescence staining of α-SMA protein on both surface layers (~90μm thickness on each side), middle core (interstitial tissue) regions (~400 μm thickness) of the valve; 1^st^ row: Static Controls; 2^nd^ row: Flex; 3^rd^ row: Flow; 4^th^ row: Flex-Flow conditioning; 5^th^ row: porcine heart valve as Positive control. Among the experimental groups, α-SMA-expressing cells were found to be predominant within the interstitial region (middle layer) of the engineered tissues in solely the Flex-Flow group; b) Quantification of positive staining (green; from images in part a) for α-SMA signal-intensity in four experimental groups; Samples exposed to flex-flow expressed a significantly higher level of positive α-SMA (p < 0.05) in comparison to the control group. PHV was treated as the positive control.

**Fig 5:**
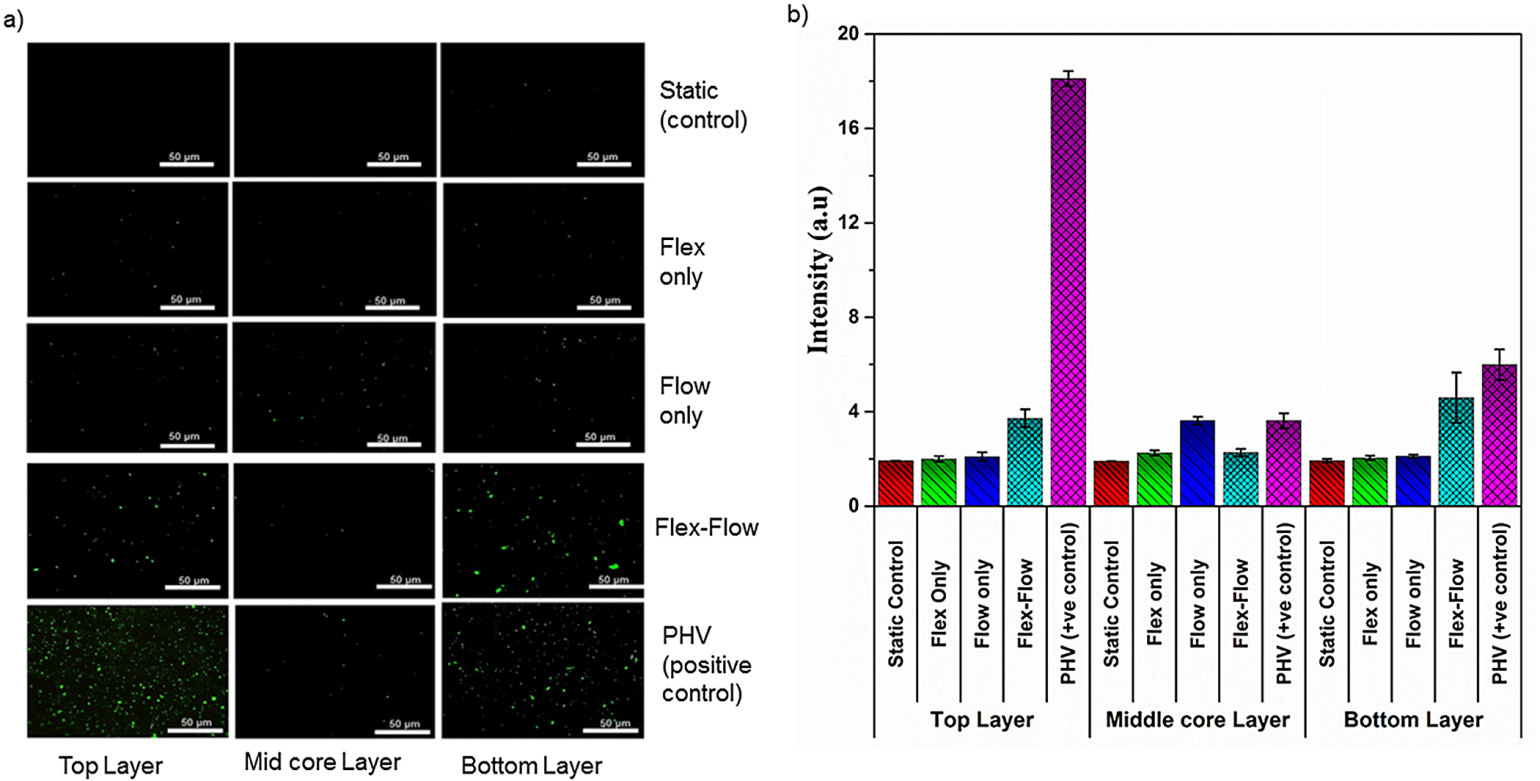
Immunofluorescence staining of CD31, an EC marker, on both surface layers (~90μm thickness on each side), middle core sections (~400 μm thickness) of the valve tissue; 1^st^ row: Static Controls; 2^nd^ row: Flex; 3^rd^ row: Flow; 4^th^ row: Flex-Flow conditioning; 5^th^ row: porcine heart valve as Positive control. Among the experimental groups, CD31-expressing cells were visible within the superficial layers (top and bottom layers) of the engineered tissues, in solely the flex flow group. b) Quantification of positive staining (green; from images in part a) for CD31signal-intensity in four experimental groups; Samples exposed to flex-flow expressed a significantly higher level of positive CD31 (p < 0.05) in comparison to the control group. PHV was treated as the positive control.

In the flex-flow group alone, α-SMA was significantly (p < 0.05) expressed in the intermediate core layer, compared to surfaces of the valve leaflets (Fig 4a and 4b). In addition, only in the flex-flow group was the endothelial marker protein CD31 abundant (p < 0.05) in the surface layers (both the top and bottom), compared to the intermediate core region of the specimens (Fig 5). All other groups produced either no expression (Static, Flexure only groups) or random distribution (flow only) of CD31 and α-SMA (S2 File).

### Gene Expression

Flex-Flow conditions suggest early evidence of supporting the valvular phenotype: Preliminary gene expression of selected markers were assessed in an effort to determine if combined Flex-Flow conditions augmented the heart valve phenotype within the in vitro grown constructs.

**Fig 6:**
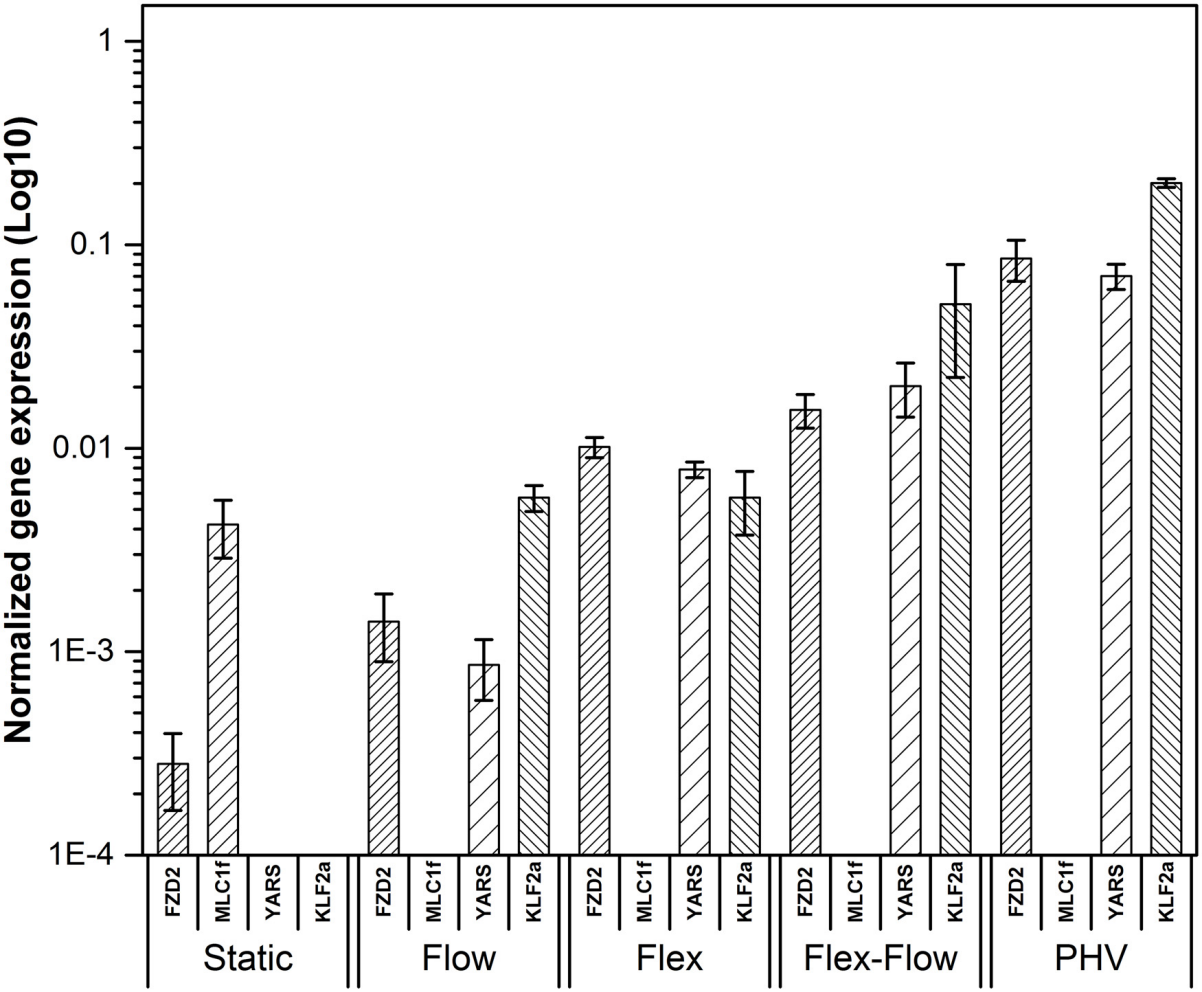
Gene expression of BMSC-derived engineered valvular tissues. The four groups investigated were: Static Controls, Flow (850 ml/min), Flex (1 Hz) and Flex-Flow (Simultaneous application of 850 ml/min flow rate and 1Hz frequency for cyclic bending of specimens). A flow rate of 850ml/min for cell culture media circulating through the bioreactor conditioning chambers permitted physiologically-relevant^4^ fluid-induced mean shear stresses of 2.91 dynes/cm^2^ and 4.73 dynes/cm^2^ on the inner and outer specimen walls respectively. PHV is treated as the positive control as a reference.

The highest level of expression for both FZD2 and YARS markers, for cardiovascular SMC (when MLC1f is not expressed) and EC related genes, respectively, was observed in RNA extracted from the flex-flow group, in which the samples were exposed to combined cyclic flexure (1 Hz) and flow (850 ml/min) conditions in the bioreactor (Fig 6); note that MLC1f was only significantly expressed in the no flow control group (P < 0.05). The KLF2A, transcription factor, critical for valvulogenesis in valve development, was also expressed significantly (p<0.05) in the flex-flow case, in comparison to the other groups investigated (no flow control, flow-alone, cyclic flexure-alone) (S2 File).

### Computational Results

Shear stress magnitudes and oscillations on the Flex-Flow cultured specimens fell within the physiological range and distribution of shear stresses for heart valves: Quantification of the fluid-induced shear stress spatial and temporal distribution on the tissue engineered specimens necessitated that a CFD simulation and subsequent analysis of the resulting data be performed.

**Fig 7:**
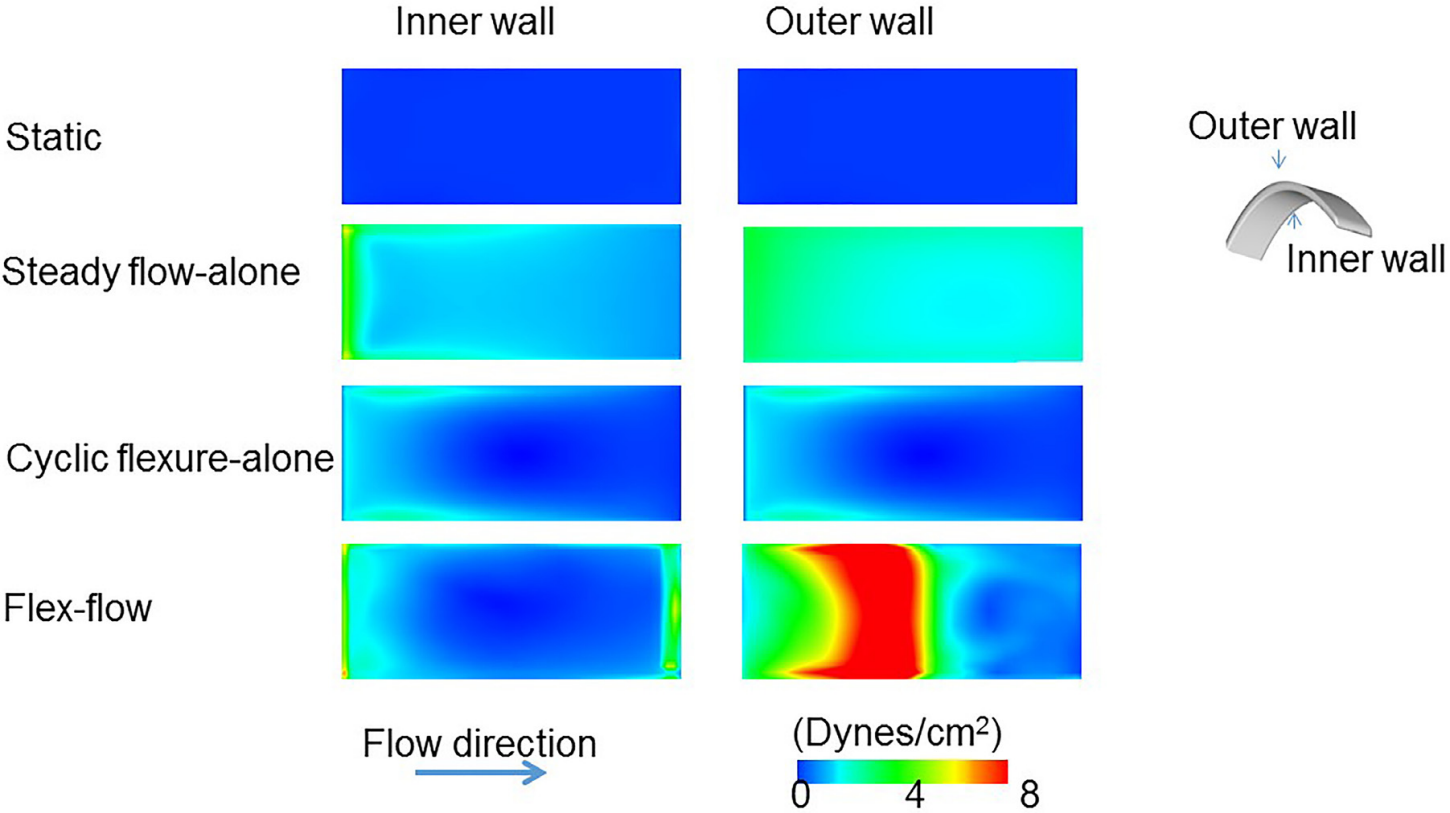
Fluid-induced, time-averaged shear stresses over one cycle on specimen inner and outer walls for the following four cases: i) Static Controls, ii) Flow, iii) Flexure, and iv) Flex-Flow states. In comparing the two dynamic cases (iii) versus (iv), the flex-flow state displayed much higher shear stress values.

The fluid-induced, time averaged shear stress magnitudes (TSSM) were plotted on the inner and outer surfaces for all the simulation cases (Fig 7). The inner wall TSSM values were 0 dyne/cm^2^, 1.98 ± 0.37 dyne/cm^2^, 0.1 ± 0.005 dyne/cm^2^, and 2.91 ± 0.11 dyne/cm^2^ for cases of static control, steady flow-alone, cyclic flexure-alone and flex-flow. The outer wall TSSM values were 0 dyne/cm^2^, 2.43 ± 0.06 dyne/cm^2^, 0.1 ± 0.003 dyne/cm^2^, and 4.73 ± 0.09 dyne/cm^2^ for cases of control, steady flow-alone, cyclic flexure-alone and flex-flow.

**Fig 8:**
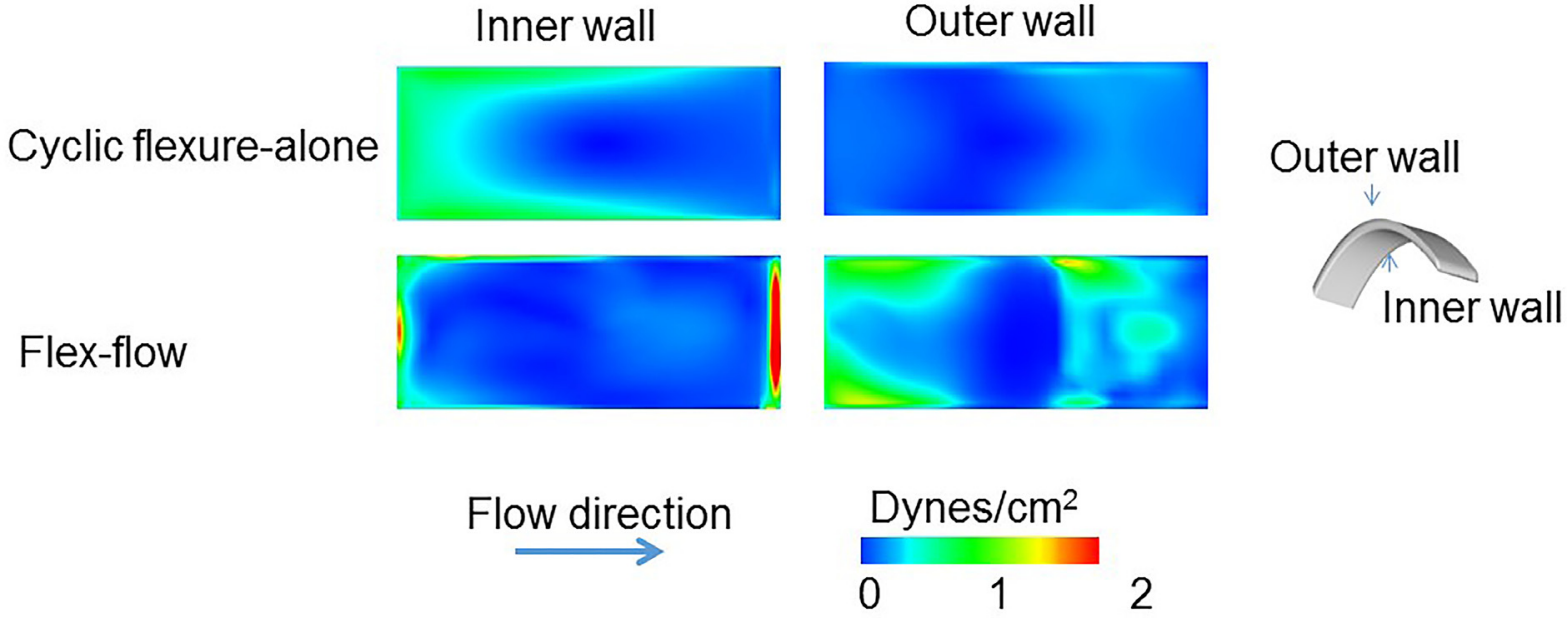
OSI-scaled shear stress magnitude (osi-$\vec{\left|\tau \right|}$) on the inner and outer specimen surface for the dynamic Flex and Flex-Flow cases.

The OSI distribution was found to be much higher when exposed to cyclic flexure-alone, relative to flex-flow conditions. The area averaged OSI on the specimen’s inner walls (n = 3) was 0.433 ± 0.015 and 0.117± 0.003 for cyclic flexure-alone and flex-flow cases, respectively, while the corresponding values for the outer wall (n = 3) were found to be 0.313± 0.011 and 0.094± 0.10. The area averaged OSI-$\vec{\left|\tau \right|}$ metric was determined to quantify the extent of coupled shear stress magnitude and temporal oscillations in the experiments performed. Over the specimen area (sample size n = 3/group), the area averaged OSI-$\vec{\left|\tau \right|}$ for the inner walls were 0.1±0.05 dyne/cm^2^ (cyclic flexure-alone) and 0.41±0.11 dyne/cm^2^ (flex-flow), while for the outer walls, it was 0.16 ±0.03 dyne/cm^2^ (cyclic flexure-alone) and 0.27±0.16 dyne/cm^2^ (flex-flow) (Fig 8). Note that OSI-$\vec{\left|\tau \right|}$ = 0 in the event that flow is temporally unidirectional or if fluid-induced shear stresses are negligible, which was the case in the steady flow-alone and no flow, respectively.

## Discussion

Functional replacement of anomalous heart valves, particularly in the case of pediatric critical valve disease, is in dire need of suitable treatment approaches; the primary limitation is that currently available replacement devices do not offer provision for somatic growth. For this reason, the notion of autologous and living biological heart valve substitutes is very appealing. Several studies have demonstrated that the process of developing robust engineered valvular tissues necessitates a dynamic culture process, wherein mechanical stresses, such as flow, flexure and stretch, are imparted onto the growing constructs [9,16,26,28,43,44]. These stress states are particularly relevant because valve leaflets experience highly varied and leaflet side-dependent, fluid-induced shear stress distributions, as well as localized cyclic tissue stretching and flexure during the cardiac cycle. In the aortic valve for example, the ventricular-side of the leaflet experiences significantly higher fluid-induced shear stresses compared to the arterial-side. Some *in vitro* investigations have been able to recapitulate important features of this hemodynamic environment using bioreactors to grow *de novo* valvular tissues [28,29,40]. However, the specific effects of *in vitro* mechanical conditioning on cell distribution and phenotype within the construct remain unclear. These attributes could support the integration of engineered to native tissues, and thus could play a critical role in guiding subsequent valve remodeling events *in vivo*. Moreover, the importance of maintaining a physiological range of conditioning parameters (e.g. fluid-induced shear stress) has also not been sufficiently addressed [19,45]. On the other hand, the utility of stem cells, such as BMSCs, has been explored in several heart valve tissue engineering approaches to date, which have demonstrated robust tissue growth under mechanical-stimulated environments [9,22,43]. Thus, in an attempt to identify the effects of valve-relevant stress environments on stem cell phenotype, as well as the implications of physiological scales of mechanical conditioning, we subjected BMSC-seeded scaffolds to individual and combined modes of fluid shear and flexural stress states, which are both highly relevant to valvular tissues.

We found a significant (p<0.05) increase in the BMSC-derived collagen content in the flex-flow group relative to the other groups, whereas no significant differences were found between the Static Control, Flex and Flow groups (Fig 2). Our observation was consistent with previous findings (Engelmayr et al.) [9] where flex-flow studies under sub-physiological shear stresses (average wall shear stress = 1.15 dynes/cm^2^) [26] were combined with physiological levels of cyclic flexure (1Hz, which corresponds to a heart rate of 60 beats/minute). However, we attempted to recapitulate the regionally varying and side-specific nature of fluid-induced, shear stress distribution of native aortic valves on our engineered tissue specimens. Indeed, previous work [28] has demonstrated that the inner and outer walls of our specimens are analogous to the aortic and ventricular-side of the leaflets. Here, under flex-flow states, the TSSM on the outer wall was 4.73 dynes/cm^2^, while along the inner wall, the shear stress was found to be 2.91 dyne/cm^2^. The ventricular side of the native human aortic valve is exposed to a TSSM in the order of ∼ 3.87 dynes/ cm^2^ [4] and thus, the shear stresses determined for the flex-flow specimens in this study are physiologically relevant. In comparing our studies to those conducted at sub-physiologic shear stress levels [9,26] we found an additional increase in collagen production by 70%, i.e., when the specimen shear stress magnitudes and distribution were regionally similar to native aortic valve leaflets. We note that an earlier study [28] demonstrated the importance of physiological scales of conditioning by way of mimicking arterial pressure conditions *in vitro*, which ultimately resulted in ∼ 35% increase in collagen content compared to sub-physiological flow environments. We speculate that the differences were more pronounced in our case as a direct result of cell regulatory mechanisms that are known to be initiated by fluid-induced shear stresses [46–51] and which we believe to also be applicable to differentiating BMSCs.

Previous investigations have examined the through-thickness distribution of cells and phenotype in TEHVs [9,24]. Consistent with flex-flow studies that employed a sub-physiologic range of shear stress conditioning [9], we observed that endothelial cellular expression was expressed primarily on the surfaces and was negligible in the interstitial layers (Fig 4). We interpret that BMSC to endothelial cell differentiation and intra-scaffold BMSC migration patterns are enhanced under flow states and is not strongly flow magnitude-dependent. Indeed, we previously visualized and monitored the increase in cell scaffold migratory patterns via magnetic resonance imaging, which was found to be augmented under steady flow-alone conditions compared to no flow controls [52]. On the other hand, the link between the mechanical environments and α-SMA expressing cells within the *de novo* valvular tissues is not as straightforward. Engelmayr et al.[9], showed the robust expression of α-SMA-expressing BMSCs preferentially distributed on the surfaces of engineered heart valve tissues, after being subjected to flex-flow states that imparted sub-physiological magnitudes of fluid-induced shear stresses. Meanwhile, Hoestrup and colleagues [24] described a consistent distribution of α-SMA-expressing BMSCs throughout tri-leaflet engineered valvular constructs subjected to physiological pressure ranges (30 to 75 mmHg) in a bioreactor; cell density was in addition the greatest on the surface of the TEHVs, and sparse deep within the tissues. We observed that application of heart valve, physiologically relevant fluid-induced shear stresses (TSSM for, inner wall: 2.91dyne/cm^2^ and outer wall: 4.73dyne/cm^2^) served to promote cellularity, particularly, α-SMA-expressing BMSCs (Fig 4). While this occurred under both flow-alone and flex-flow conditions, it was only in the latter group where α-SMA-expressing cells were predominant within the interstitial regions of the engineered tissues and were found to be sparse on the superficial layers. On the other hand, as described earlier, CD31-expressing cells indicative of an endothelial phenotype were distributed preferentially on the surfaces of the engineered tissues specimens in only the flex-flow group (Fig 5). Even though the extent of both α-SMA and CD31 immunofluorescence staining was less pronounced than native valves, as was also evidenced for the valve-relevant markers in the gene expression outcomes, it is clear that physiological scales of flex-flow conditioning promote heterogeneous cell distribution in a manner that mimics native valve cellular distribution. We interpret the ability to achieve heterogeneous, valve-relevant, BMSC distribution and differentiation in an *in vitro* setting to play a critical role in continued tissue remodeling after implantation, including accelerated replication of the native tri-layered valves structure. Such a structure has only been observed in Ovine studies after several weeks (16–32 weeks) [53] following TEHV implantation, and has been limited to solely the replacement of the pulmonary valve rather than the more demanding aortic or mitral positions. Thus, in specific cases of heart valve diseases, such as for example, in the treatment of critical congenital aortic valve stenosis in infants [54–58], accelerated tissue remodeling may be especially important in order to keep pace with somatic growth.

As further evidence of valvulogenesis, we found that flex-flow conditioning significantly augmented (p < 0.05) the KLF2A gene compared to all other groups (Fig 6). In native valve development, the absence of KLF2A leads to substantial valve malformations and is directly modulated by oscillatory fluid-induced shear stresses [11]. Interestingly, we were able to demonstrate that the upregulated expression of KLF2A by stem cells rather than endogenous valvular cells, specifically BMSCs, is also possible when subjected to flex-flow conditions. Our previous work showed that OSS are enhanced under flex-flow conditions and hence are likely to have elicited cellular upregulation of KLF2A.

When making collective comparisons of KLF2A markers expression observed in the flex-flow group relative to corresponding native valve gene expression, we found not surprisingly, that native valve expression was higher, in the order of 49%. The marked lower density of BMSCs in time-limited, *in vitro* culture environments is likely to have exhibited truncated gene expression levels compared to gene expression of cells obtained from native valvular tissues. The broader argument that needs to be considered however, is that the promotion of the valvular phenotype *in vitro*, even if it is small, may be critical in enhancing integration between engineered to native valvular tissues after implantation. From a phenotypic viewpoint, the recognition of the engineered construct by its surrounding *in vivo* cellular environment is likely to be critical in paracrine cell signaling events, which will serve to ensure guided remodeling of the TEHV and minimize the risk of tissue overgrowth and uncontrolled pannus. Previous studies have already demonstrated the importance of recapitulating the native tri-layer tissue structure in TEHVs in support of robust functionality [53,59]. Similarly, we speculate that the phenotypic state of BMSC-derived engineered constructs is equally important prior to implantation, which we show here to be directed toward the valvular lineage under flex-flow mechanical conditioning. Thus, we interpret that flex-flow mechanical conditioning during *in vitro* culture of engineered tissues supports BMSC differentiation in a heterogeneous manner, i.e., by promoting both the valvular endothelial and interstitial cell phenotypes. This finding corroborates our previous computational predictions that associated the simultaneous presence of both OSS and the critical magnitude of shear stress during flex-flow states to synergistically increase collagen content in the engineered tissues [40], which appears to also enhance, as evidenced here, the valve phenotype.

In summary, physiologically relevant flex-flow states serve to promote cell distribution and phenotype in *in vitro* grown, BMSC-derived, engineered heart valve tissues. We were able to demonstrate for the first time that BMSC differentiation and migration within engineered tissues led to surface-lined endothelial-marker expressing cells and interstitium-filled, myofibroblast-marker expressing cells, thereby resembling native valve cellular distribution. In addition, the concomitance of OSS and critical levels of shear stress enabled the robust expression of very early stages of key valvular genes, notably KLF2A [11]. Note that flex states-alone induce OSS but have negligible shear stress magnitudes and were found to not augment the cellularity and phenotype within the engineered tissues; this is consistent with our previous findings [40] in both temporal directionality and the magnitude of shear stress is important in eliciting BMSC responses, which we showed here can be achieved experimentally under flex-flow states. We conclude that the *in vitro* optimization of BMSC-derived, engineered heart valve cellular make-up and phenotype is achievable through flex-flow conditions of physiological relevance. The ability to stimulate heterogeneous valvular cellularity and phenotype is likely to be important in guiding very early stages of valve cell distribution and hence subsequent *in vivo* valve tissue remodeling events towards ensuring long-term success of the TEHV in pediatric patients.

## Supporting Information

S1 File Bioreactor setup. (PPTX)

S2 File Experimental Raw Data. (XLSX)

S3 File Purity of RNA. (PDF)

S4 File QRTPCR Melt Curve. (PDF)

S5 File CFD Set up. (PPTX)
