# Supplementary material for: Differentiation and Distribution of Marrow Stem Cells in Flex-Flow Environments Demonstrate Support of the Valvular Phenotype
Source: PLoS One. 2015 Nov 4;10(11):e0141802. doi: 10.1371/journal.pone.0141802 (PMC4633293; doi:10.1371/journal.pone.0141802)
Supplement: S1 File — (PPTX) [file pone.0141802.s001.pptx]

## Slide 1
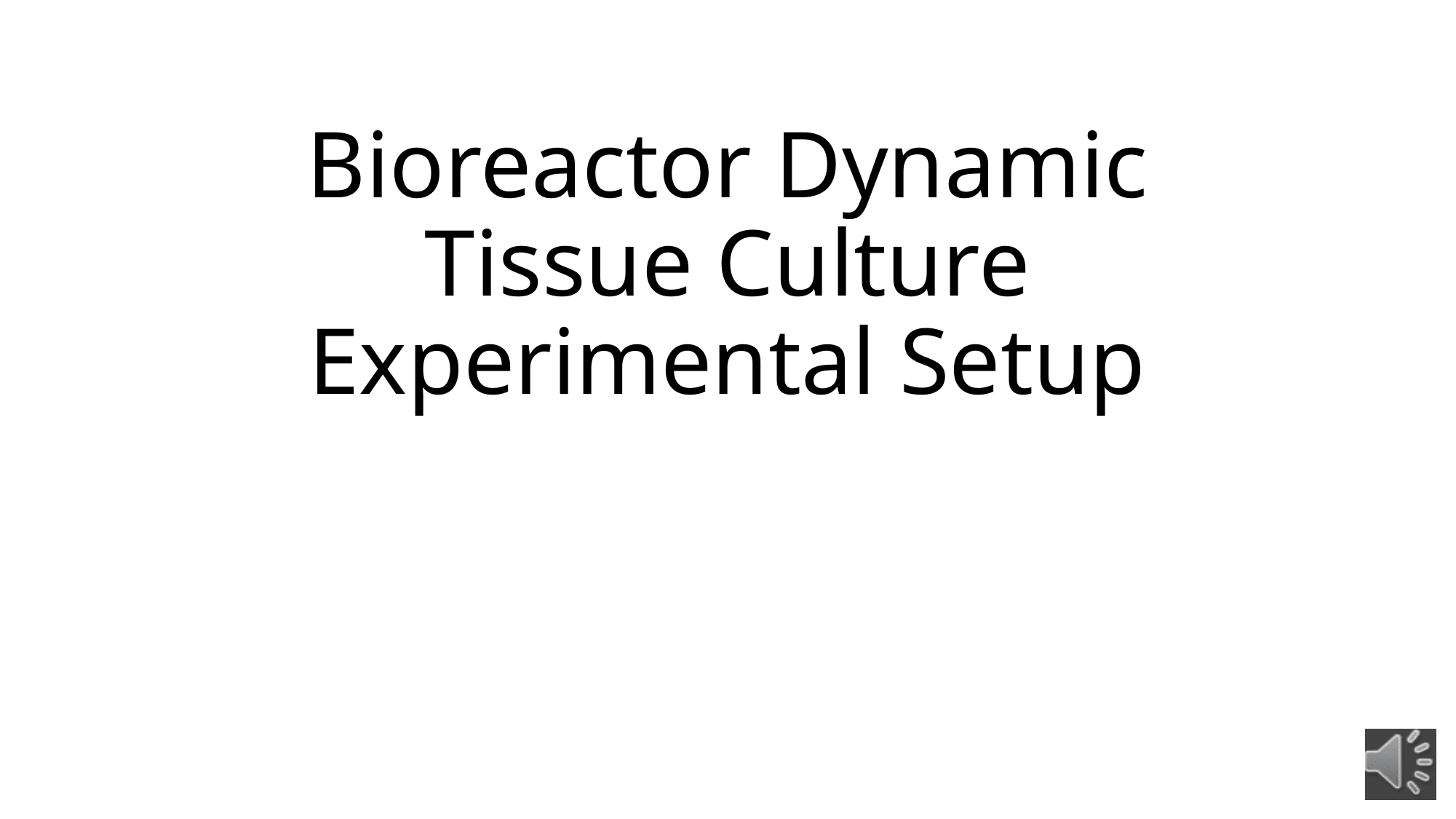

# Bioreactor Dynamic Tissue Culture Experimental Setup

## Slide 2
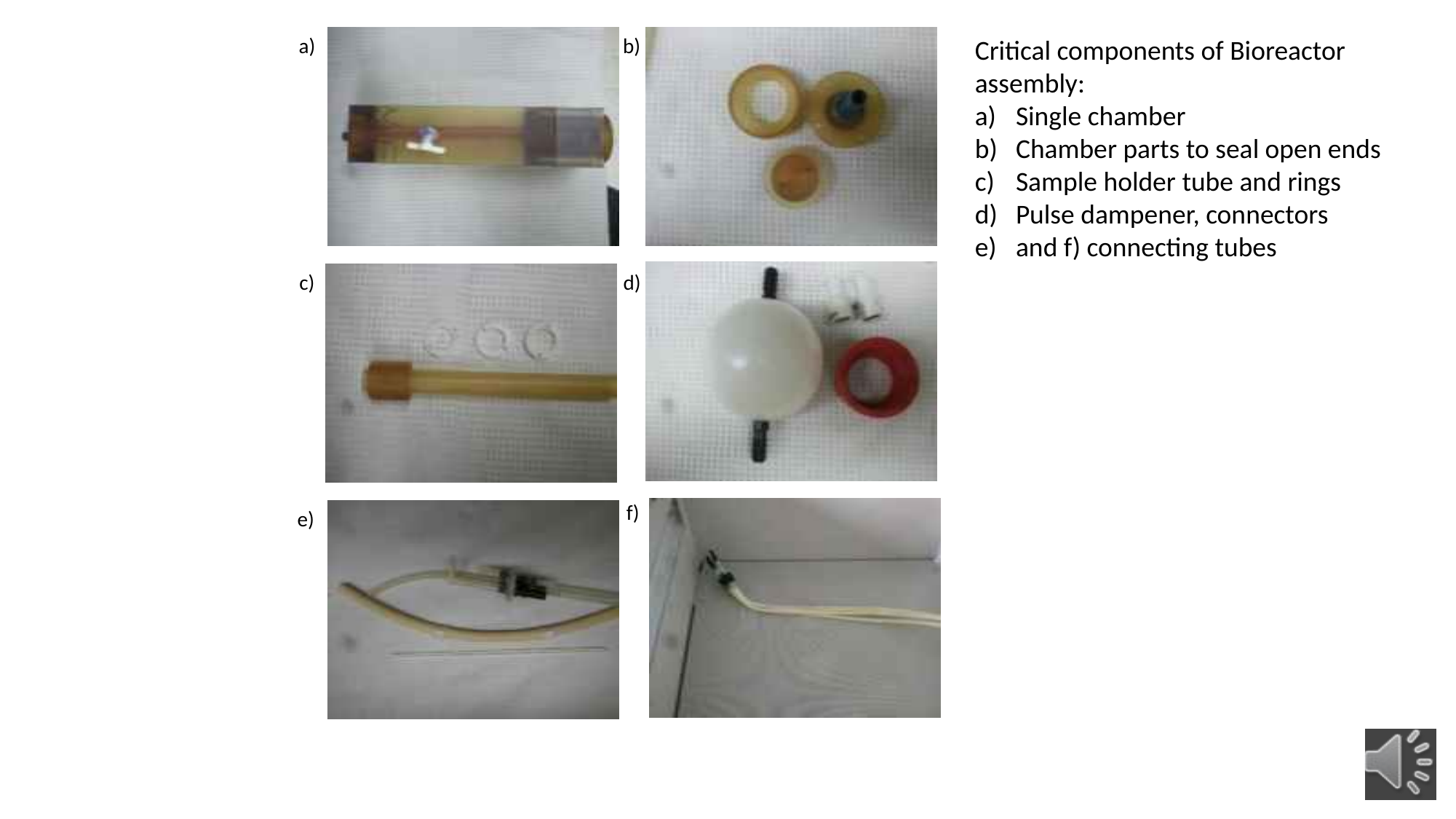

a)
b)
Critical components of Bioreactor assembly:
Single chamber
Chamber parts to seal open ends
Sample holder tube and rings
Pulse dampener, connectors
and f) connecting tubes
c)
d)
f)
e)

## Slide 3
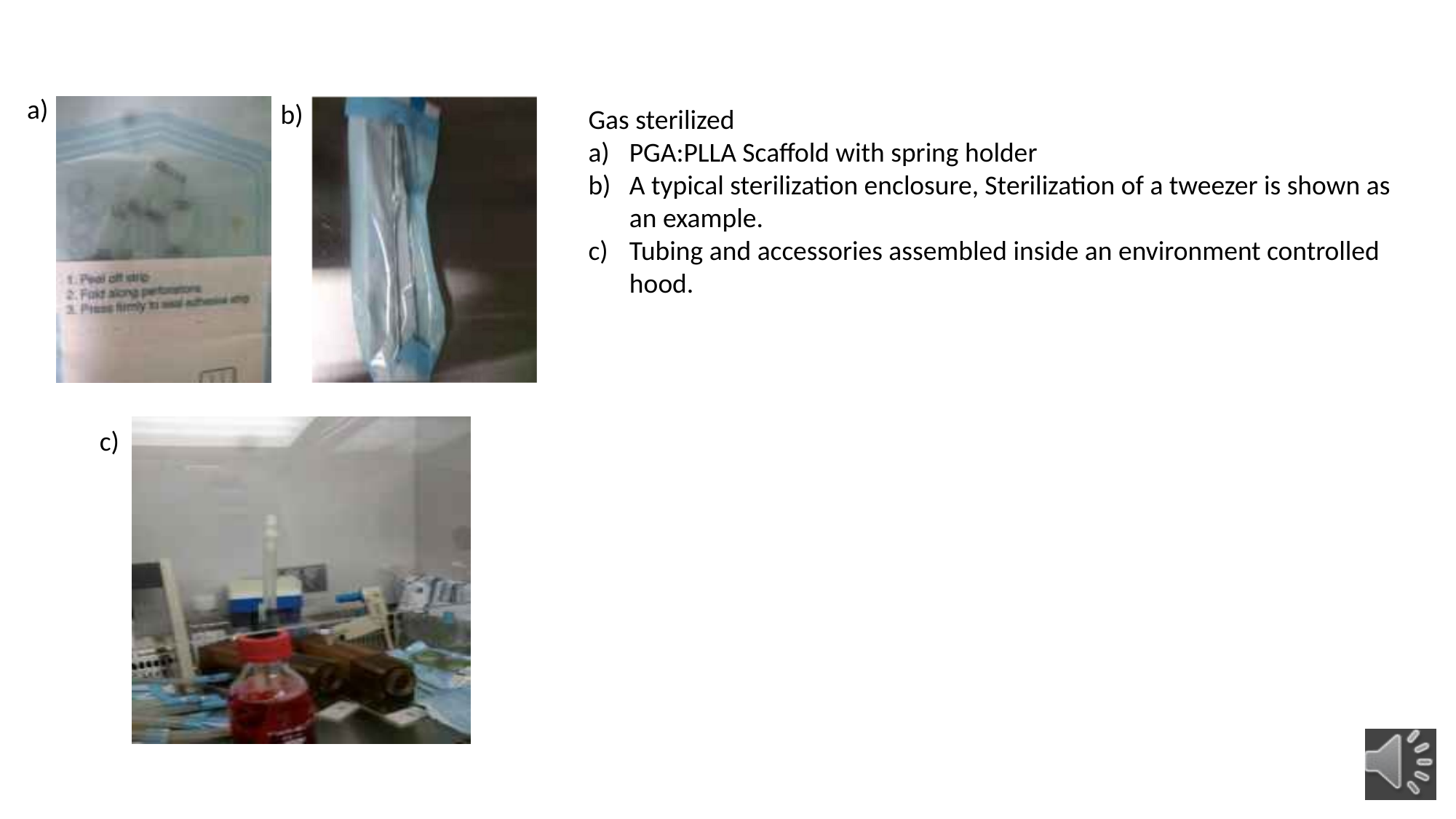

a)
b)
Gas sterilized
PGA:PLLA Scaffold with spring holder
A typical sterilization enclosure, Sterilization of a tweezer is shown as an example.
Tubing and accessories assembled inside an environment controlled hood.
c)

## Slide 4
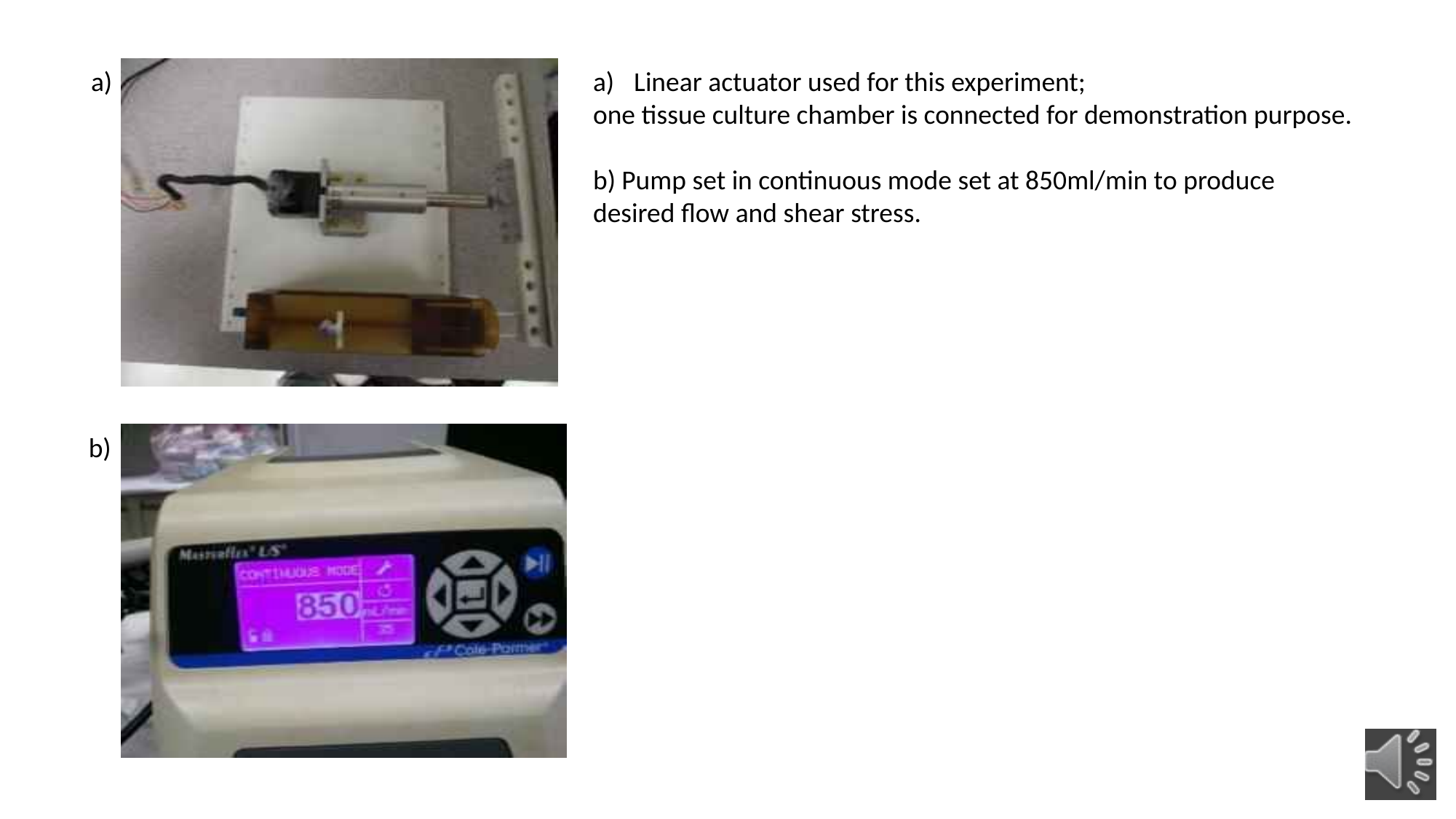

a)
Linear actuator used for this experiment;
one tissue culture chamber is connected for demonstration purpose.
b) Pump set in continuous mode set at 850ml/min to produce
desired flow and shear stress.
b)

## Slide 5
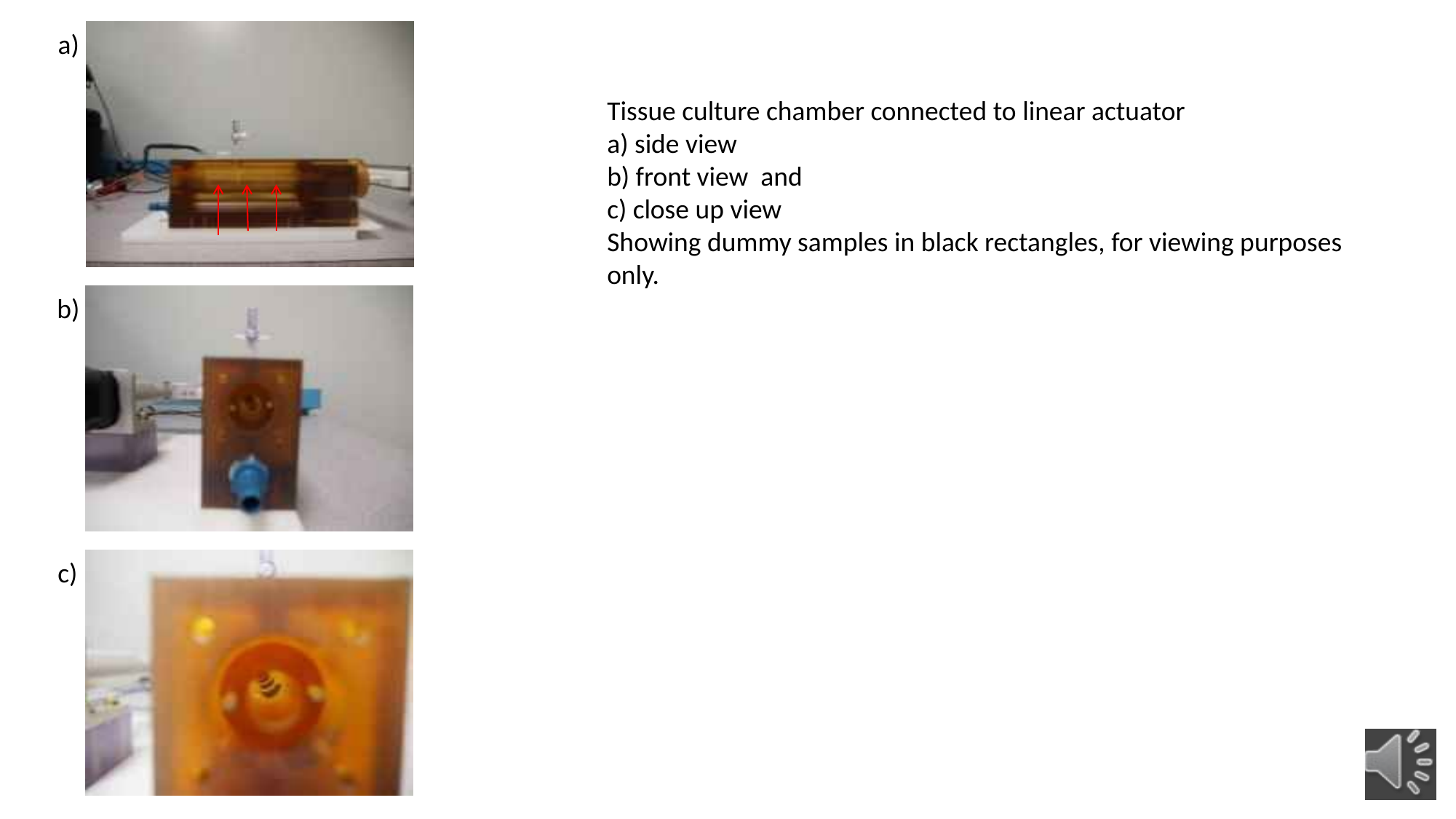

a)
Tissue culture chamber connected to linear actuator
a) side view
b) front view and
c) close up view
Showing dummy samples in black rectangles, for viewing purposes only.
b)
c)

## Slide 6
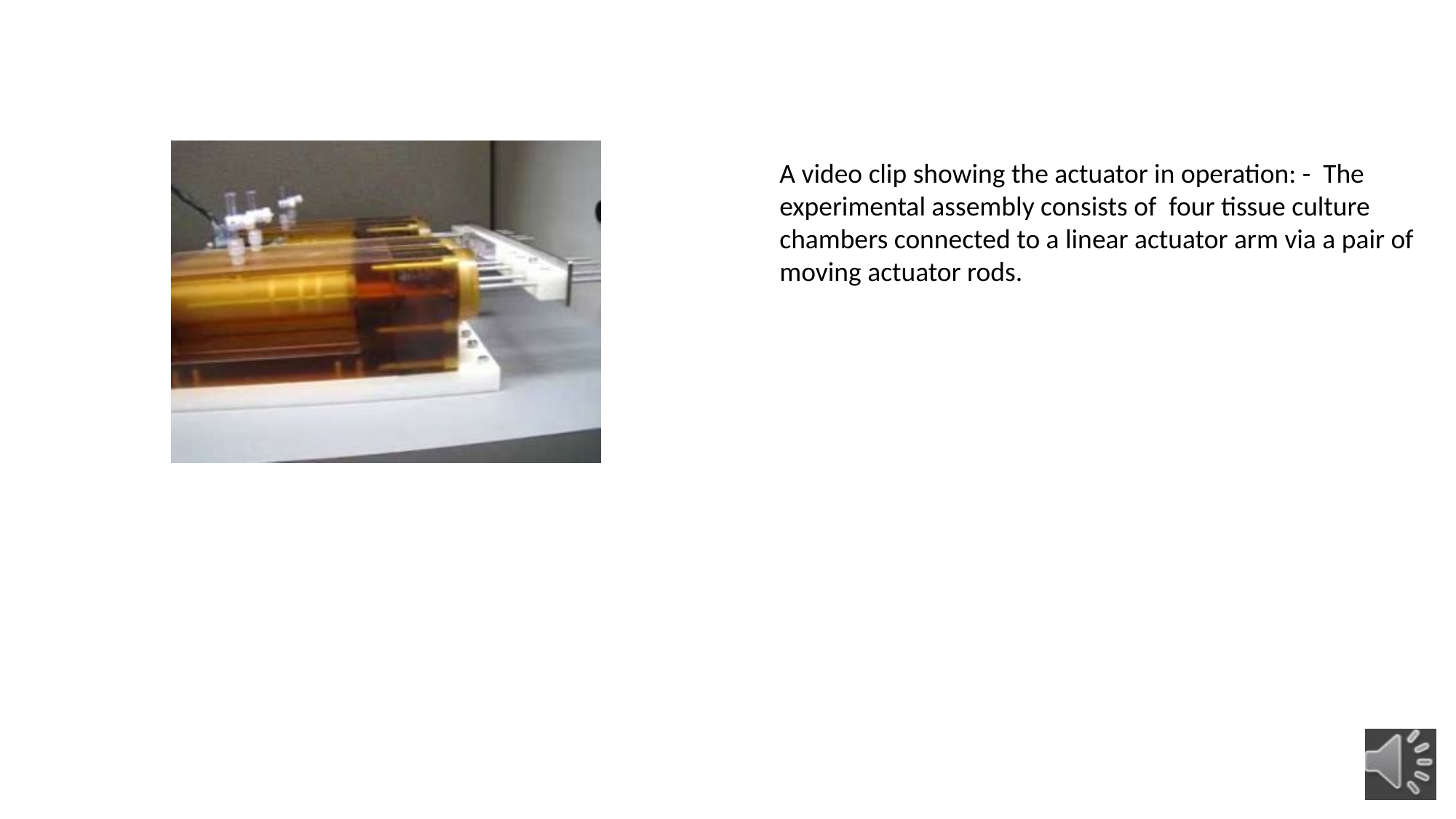

A video clip showing the actuator in operation: - The experimental assembly consists of four tissue culture chambers connected to a linear actuator arm via a pair of moving actuator rods.

## Slide 7
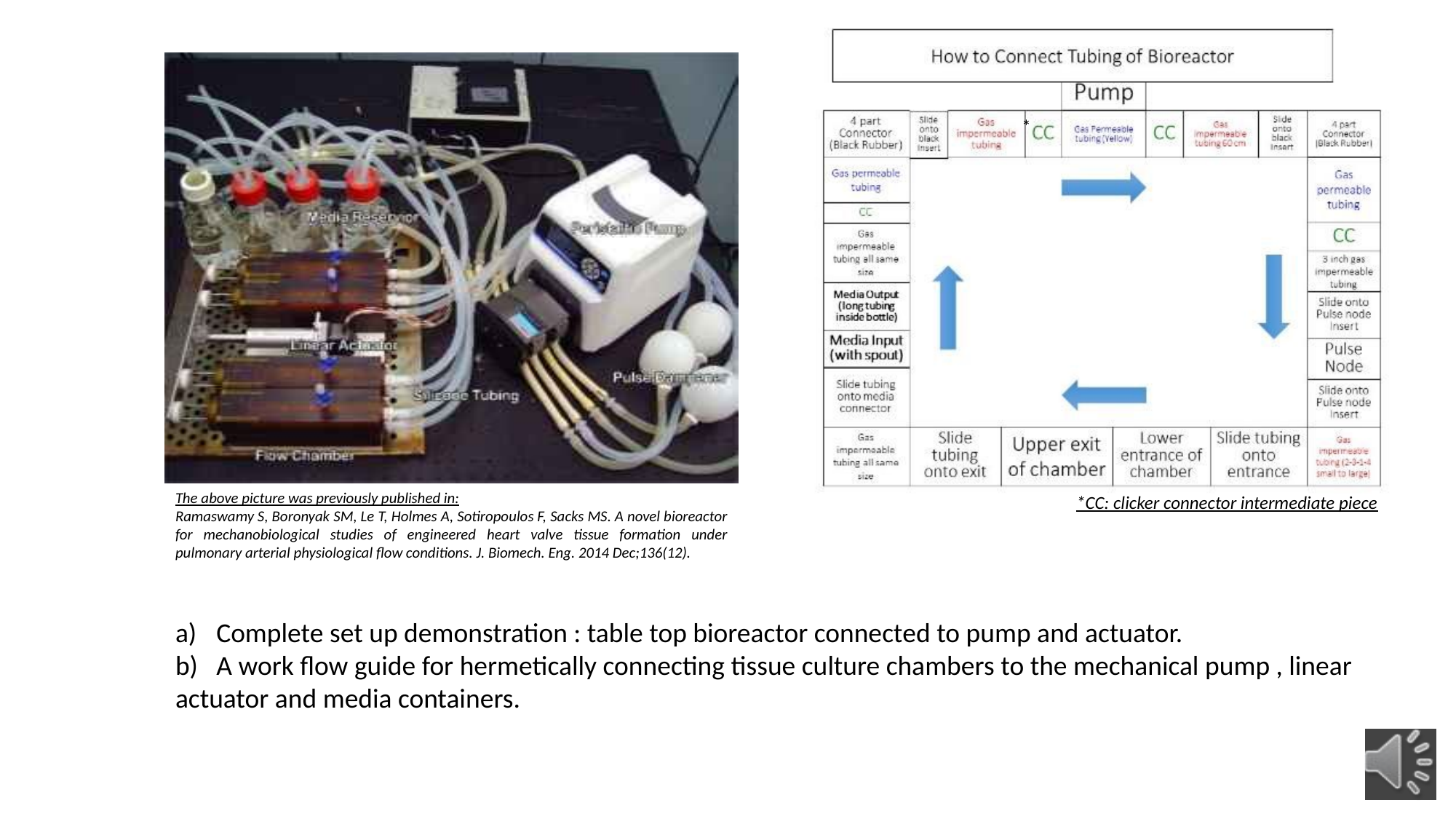

*
The above picture was previously published in:
Ramaswamy S, Boronyak SM, Le T, Holmes A, Sotiropoulos F, Sacks MS. A novel bioreactor for mechanobiological studies of engineered heart valve tissue formation under pulmonary arterial physiological flow conditions. J. Biomech. Eng. 2014 Dec;136(12).
*CC: clicker connector intermediate piece
Complete set up demonstration : table top bioreactor connected to pump and actuator.
b) A work flow guide for hermetically connecting tissue culture chambers to the mechanical pump , linear actuator and media containers.

## Slide 8
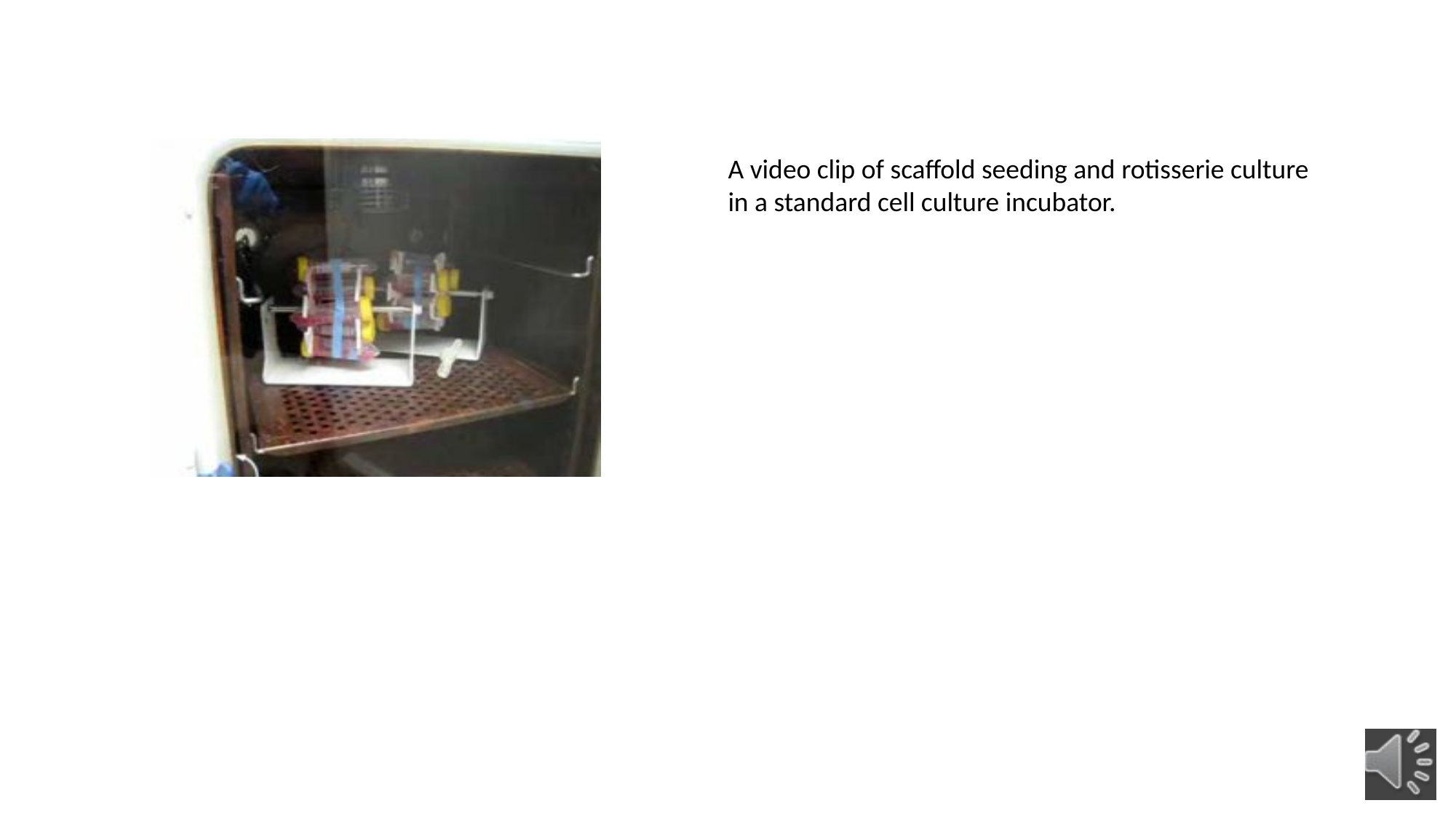

A video clip of scaffold seeding and rotisserie culture
in a standard cell culture incubator.

## Slide 9
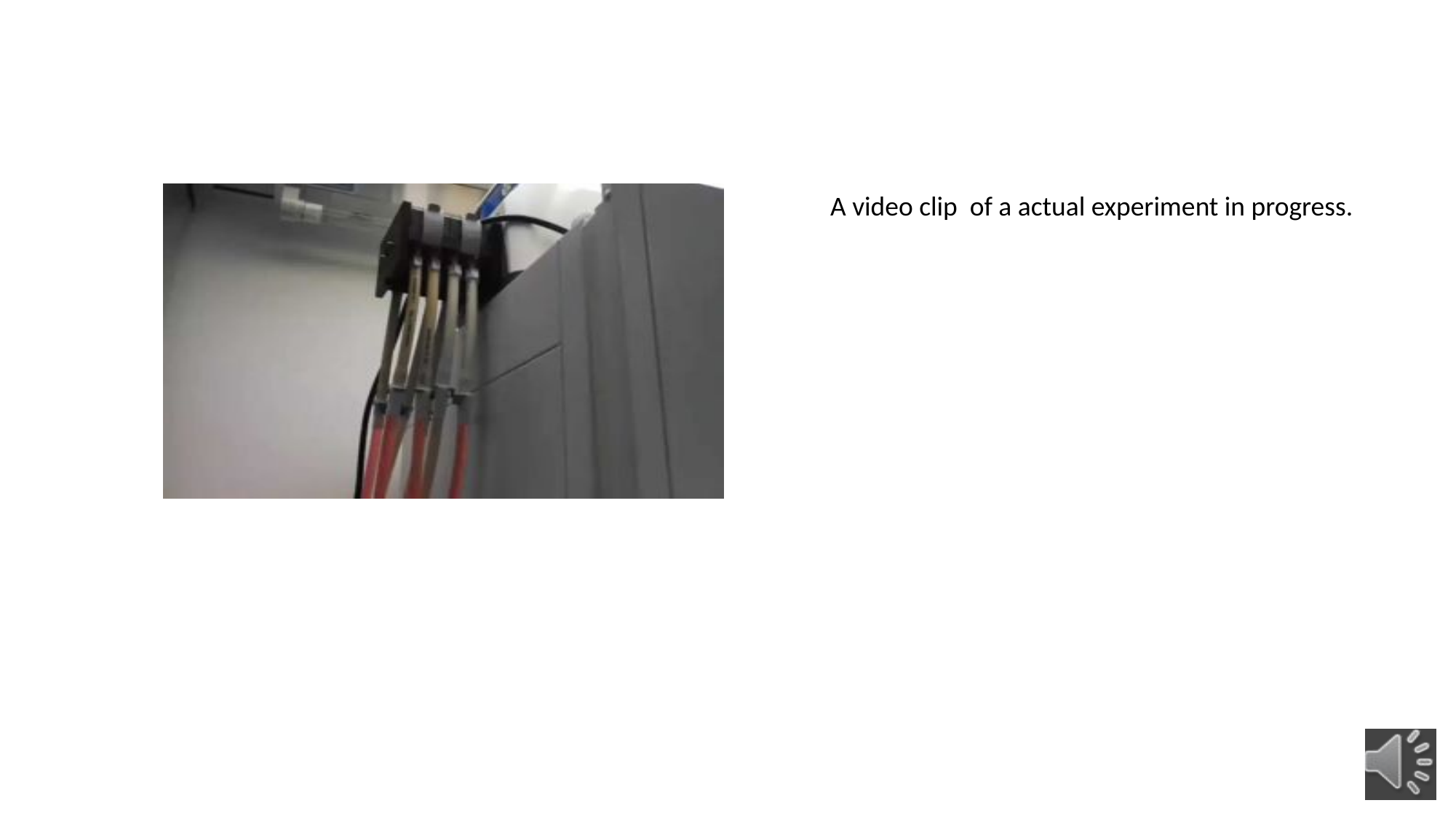

A video clip of a actual experiment in progress.
