# Supplementary material for: Differentiation and Distribution of Marrow Stem Cells in Flex-Flow Environments Demonstrate Support of the Valvular Phenotype
Source: PLoS One. 2015 Nov 4;10(11):e0141802. doi: 10.1371/journal.pone.0141802 (PMC4633293; doi:10.1371/journal.pone.0141802)
Supplement: S3 File — (PDF) [file pone.0141802.s003.pdf]

Purity of RNA

Static

RNA - DNA Report

Report time	2/4/2014 11:40:05 AM
Batch name	C:\Documents and Settings\vbhar002\Desktop\2_4_14_SR_AV.BDN
Application	RNA - DNA Estimation 3.00(184)
Operator	

Instrument Settings

Instrument	Cary 300
Instrument version no.	9.00
Ordinate Mode	Abs
SBW (nm)	2.0
Ave Time (sec)	1.000
Beam mode	Double auto select
Beam interchange	Normal
Replicates	OFF
Wavelength 1 (nm)	260.00
Wavelength 2 (nm)	280.00
Background Correction	OFF

Ratio = (A[260.00])/(A[280.00])

Analysis

Collection time 2/4/2014 11:40:05 AM

Sample	A[260]	A[280]	Ratio
Static 1	0.0729	0.0353	2.0661

Flex

RNA - DNA Report

Report time	3/10/2014 09:07:44 AM
Batch name	C:\Documents and Settings\vbhar002\Desktop\3_10_14_SR_AV.BDN
Application	RNA - DNA Estimation 3.00(184)
Operator	

Instrument Settings

Instrument	Cary 300
Instrument version no.	9.00
Ordinate Mode	Abs
SBW (nm)	2.0
Ave Time (sec)	1.000
Beam mode	Double auto select
Beam interchange	Normal
Replicates	OFF
Wavelength 1 (nm)	260.00
Wavelength 2 (nm)	280.00
Background Correction	OFF

Ratio = (A[260.00])/(A[280.00])

Analysis

Collection time 3/10/2014 09:07:44 AM

Sample	A[260]	A[280]	Ratio
Flex	0.0703	0.0353	1.9915

Flow

RNA - DNA Report

Report time	4/5/2014 01:04:15 PM
Batch name	C:\Documents and Settings\vbhar002\Desktop\Flow_4514.BDN
Application	RNA - DNA Estimation 3.00(184)
Operator	

Instrument Settings

Instrument	Cary 300
Instrument version no.	9.00
Ordinate Mode	Abs
SBW (nm)	2.0
Ave Time (sec)	1.000
Beam mode	Double auto select
Beam interchange	Normal
Replicates	OFF
Wavelength 1 (nm)	260.00
Wavelength 2 (nm)	280.00
Background Correction	OFF

Ratio = (A[260.00])/(A[280.00])

Analysis

Collection time 4/5/2014 01:04:15 PM

Sample	A[260]	A[280]	Ratio
Sample 1	0.0881	0.0466	1.8909

Flex-Flow

RNA - DNA Report

Report time	5/15/2014 03:44:12 PM
Batch name	C:\Documents and Settings\vbhar002\Desktop\F-F_SR_AV.BDN
Application	RNA - DNA Estimation 3.00(184)
Operator	

Instrument Settings

Instrument	Cary 300
Instrument version no.	9.00
Ordinate Mode	Abs
SBW (nm)	2.0
Ave Time (sec)	1.000
Beam mode	Double auto select
Beam interchange	Normal
Replicates	OFF
Wavelength 1 (nm)	260.00
Wavelength 2 (nm)	280.00
Background Correction	OFF

Ratio = (A[260.00])/(A[280.00])

Analysis

Collection time 5/15/2014 03:44:12 PM

Sample	A[260]	A[280]	Ratio
Sample 1	0.0637	0.0302	2.1125

PHV

RNA - DNA Report

Report time	6/19/2015 11:49:30 AM
Batch name	C:\Documents and Settings\vbhar002\Desktop\PHV_61915_SR.BDN
Application	RNA - DNA Estimation 3.00(184)
Operator	

Instrument Settings

Instrument	Cary 300
Instrument version no.	9.00
Ordinate Mode	Abs
SBW (nm)	2.0
Ave Time (sec)	1.000
Beam mode	Double auto select
Beam interchange	Normal
Replicates	OFF
Wavelength 1 (nm)	260.00
Wavelength 2 (nm)	280.00
Background Correction	OFF

Ratio = (A[260.00])/(A[280.00])

Analysis

Collection time 6/19/2015 11:19:30 AM

Sample	A[260]	A[280]	Ratio
PHV 111	0.0500	0.0259	1.9307
