# Supplementary figures and images for: Differentiation and Distribution of Marrow Stem Cells in Flex-Flow Environments Demonstrate Support of the Valvular Phenotype
Source: PLoS One. 2015 Nov 4;10(11):e0141802. doi: 10.1371/journal.pone.0141802 (PMC4633293; doi:10.1371/journal.pone.0141802)

RT PCR Melt curve

# Static

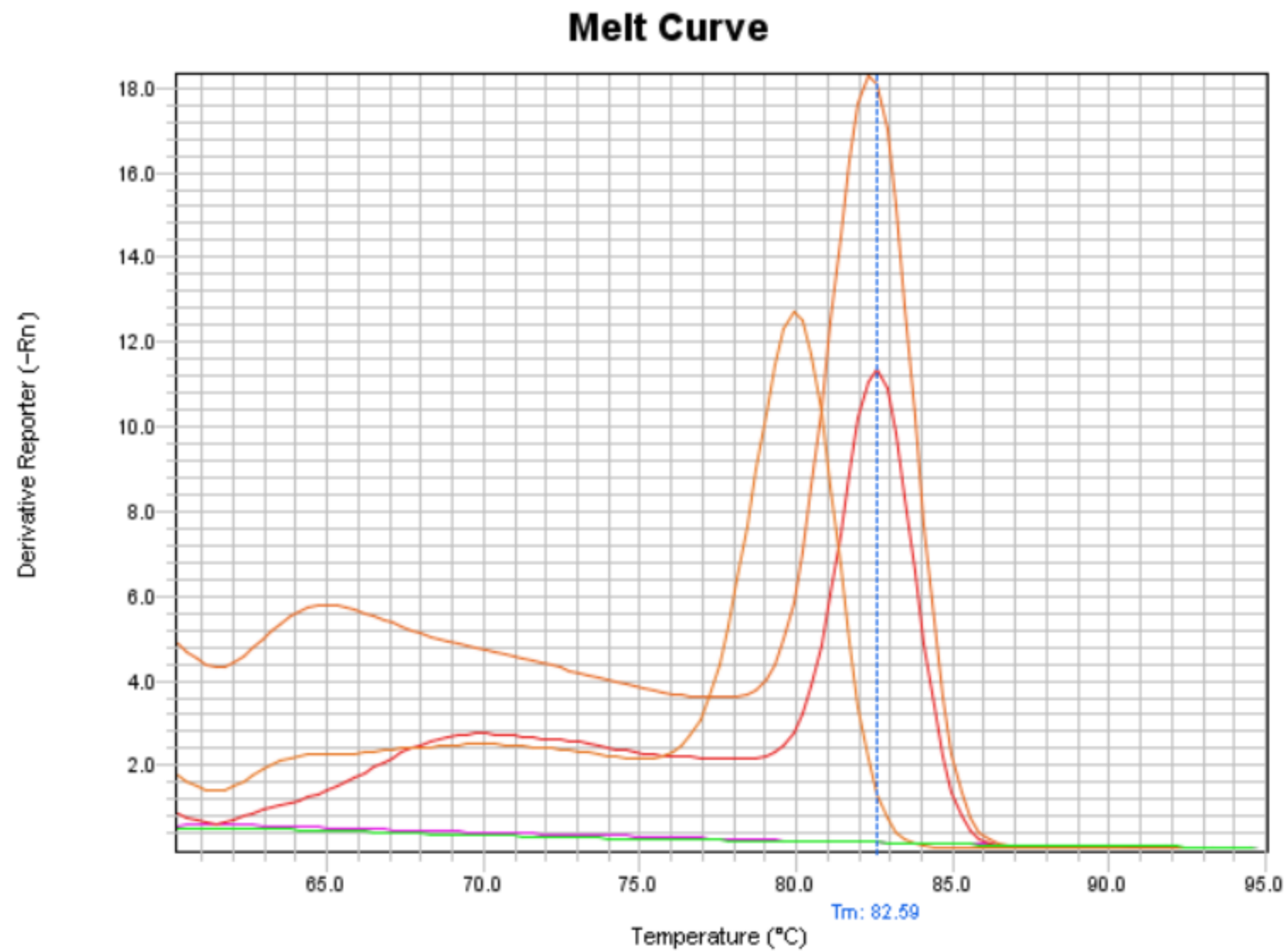

# Flow

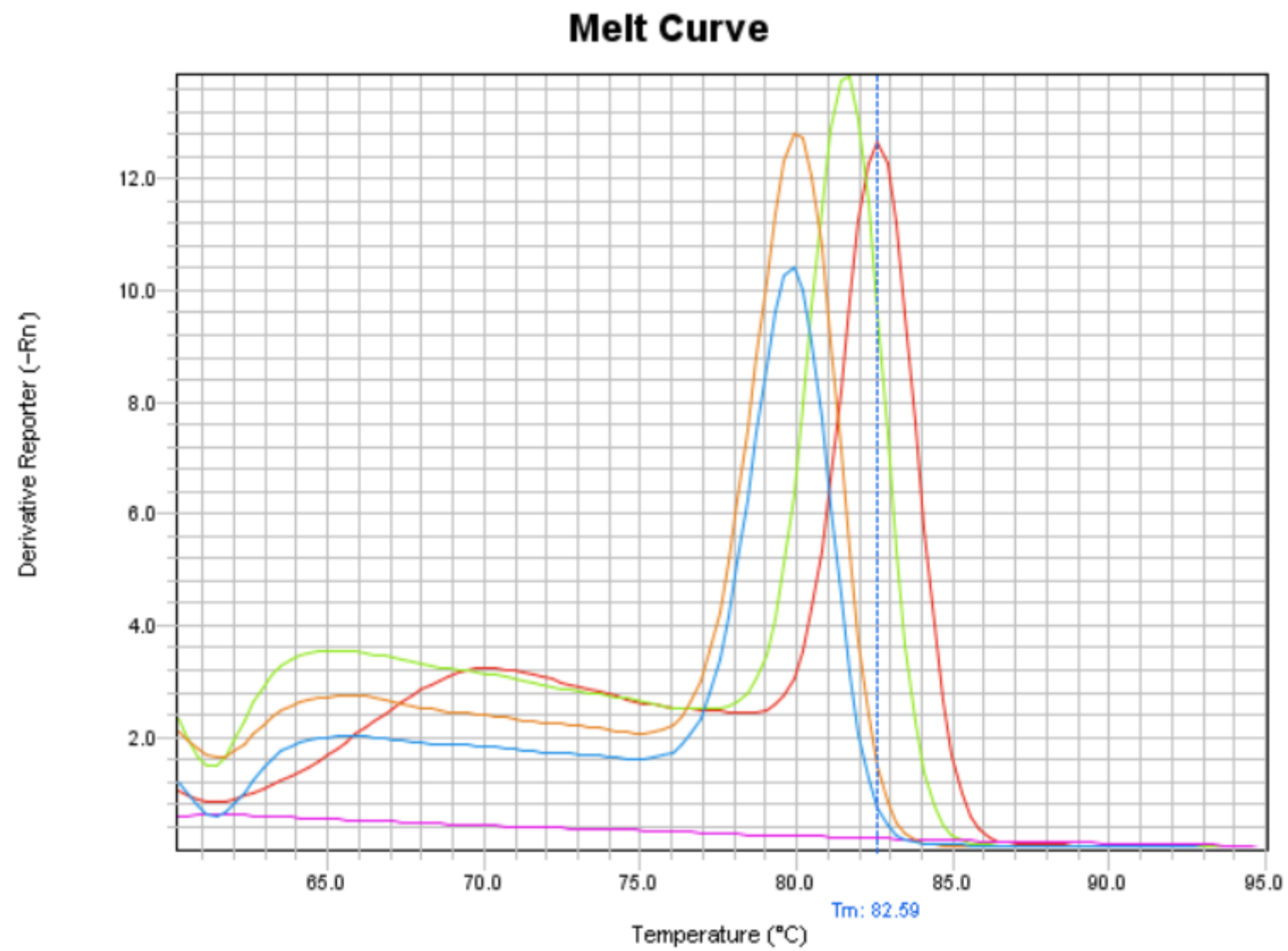

# Flex

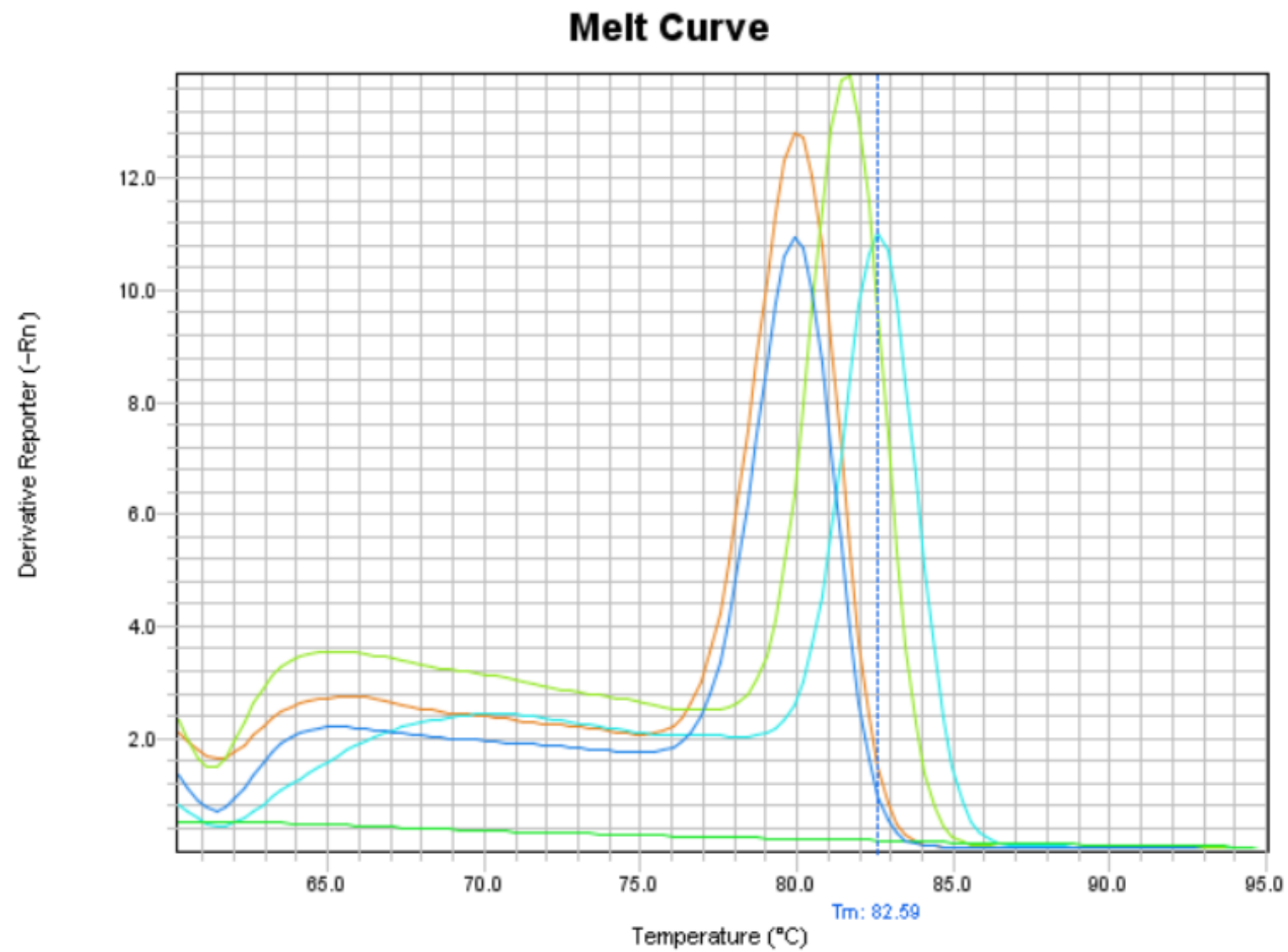

# Flex-Flow

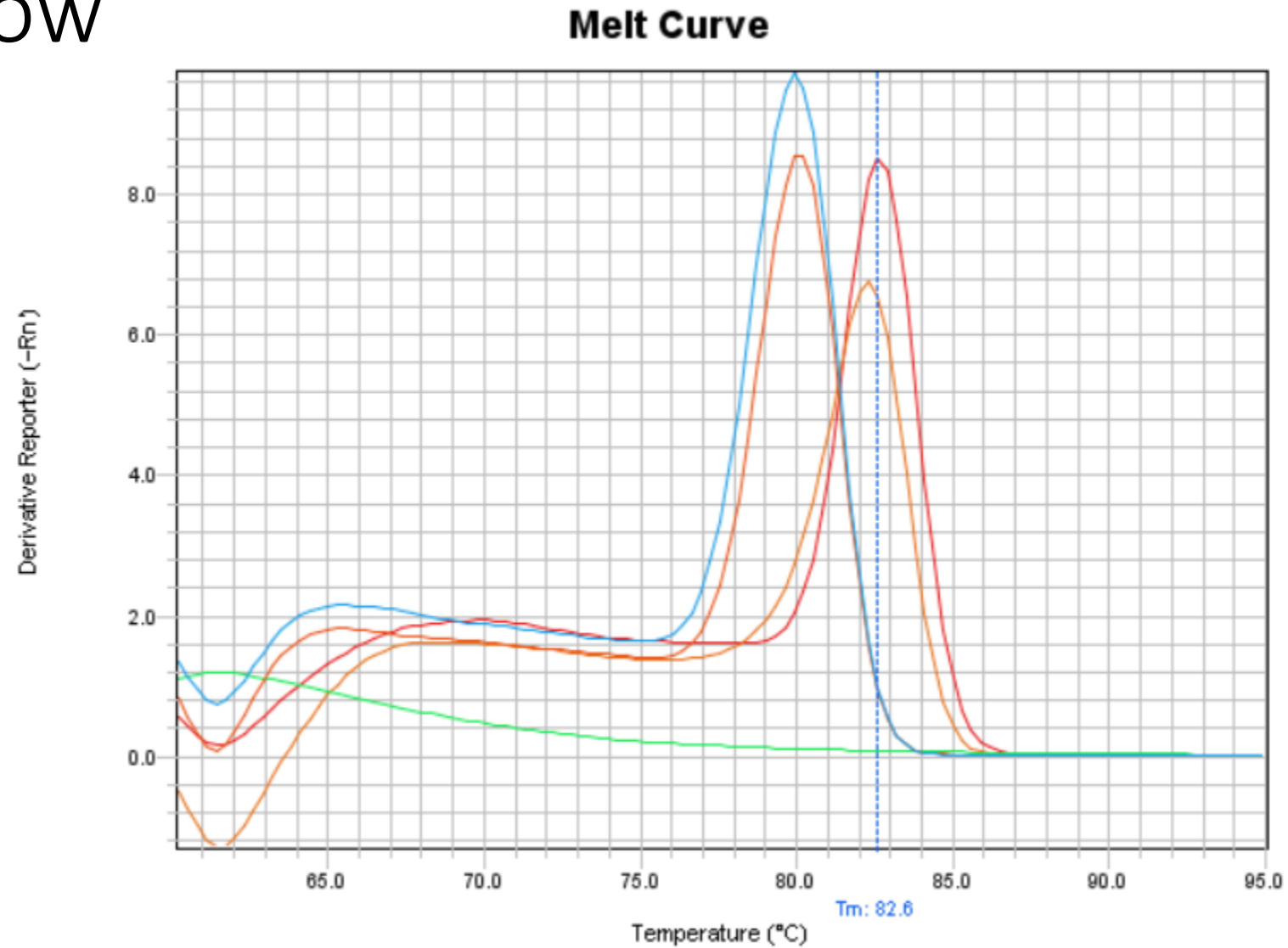

# PHV

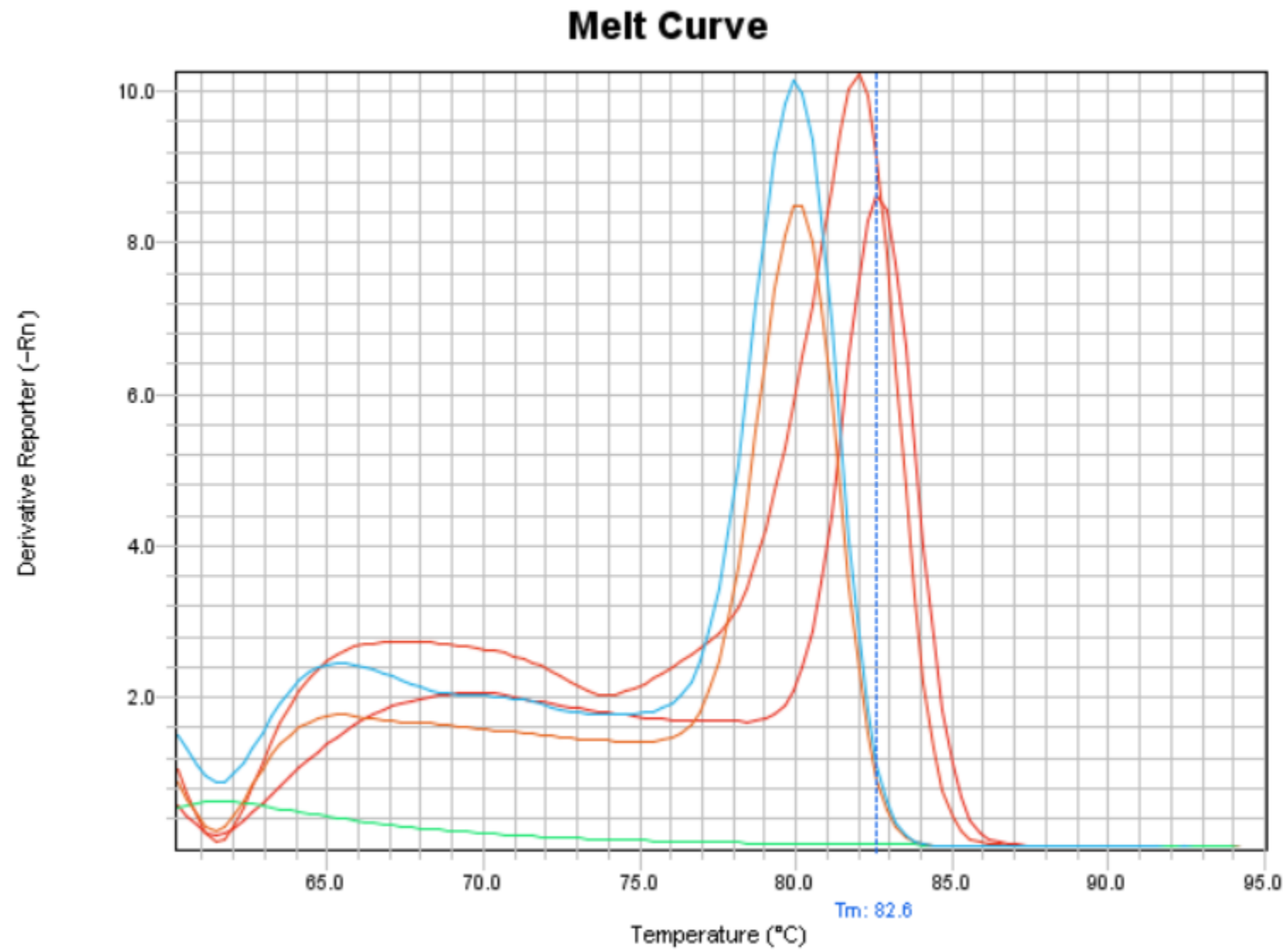

Supplement: S4 File — (PDF) [file pone.0141802.s004.pdf]
