# Supplementary material for: Differentiation and Distribution of Marrow Stem Cells in Flex-Flow Environments Demonstrate Support of the Valvular Phenotype
Source: PLoS One. 2015 Nov 4;10(11):e0141802. doi: 10.1371/journal.pone.0141802 (PMC4633293; doi:10.1371/journal.pone.0141802)
Supplement: S5 File — (PPTX) [file pone.0141802.s005.pptx]

## Slide 1
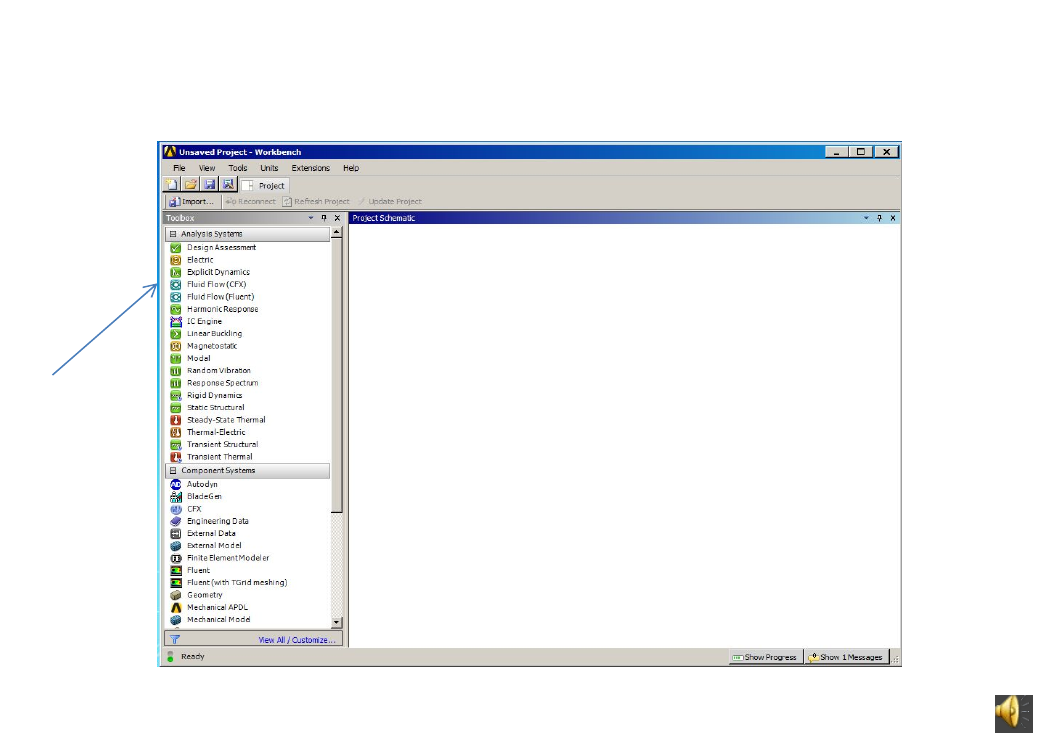

## Slide 2
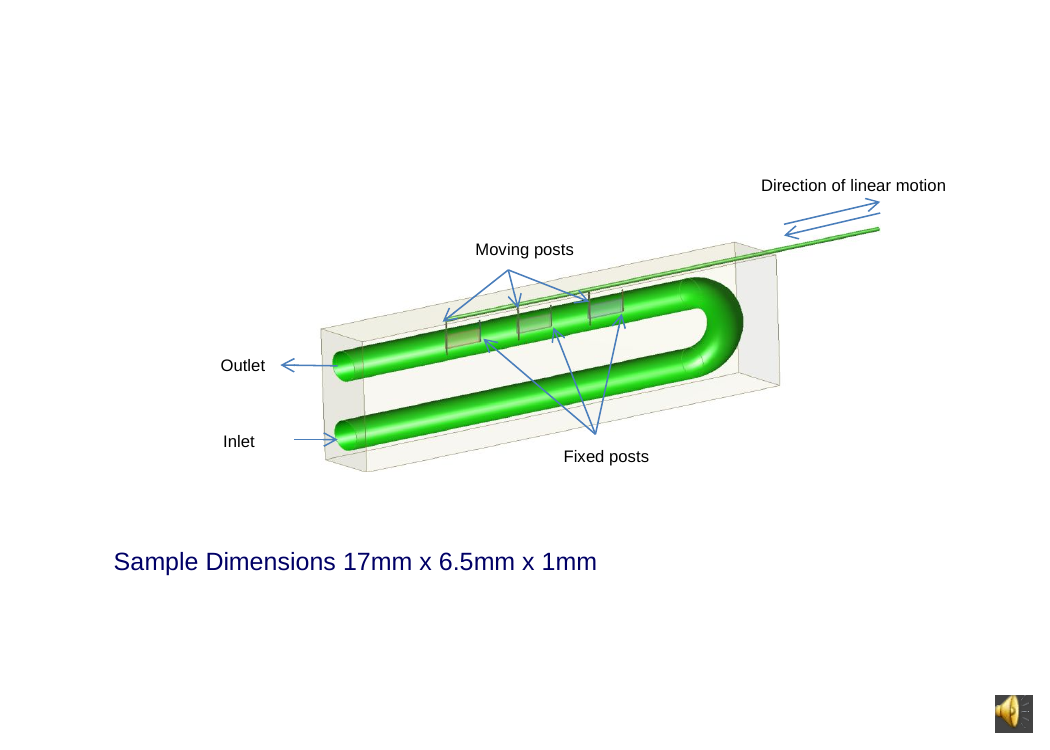

Direction of linear motion
Moving posts
Outlet
Inlet
Fixed posts
Sample Dimensions 17mm x 6.5mm x 1mm

## Slide 3
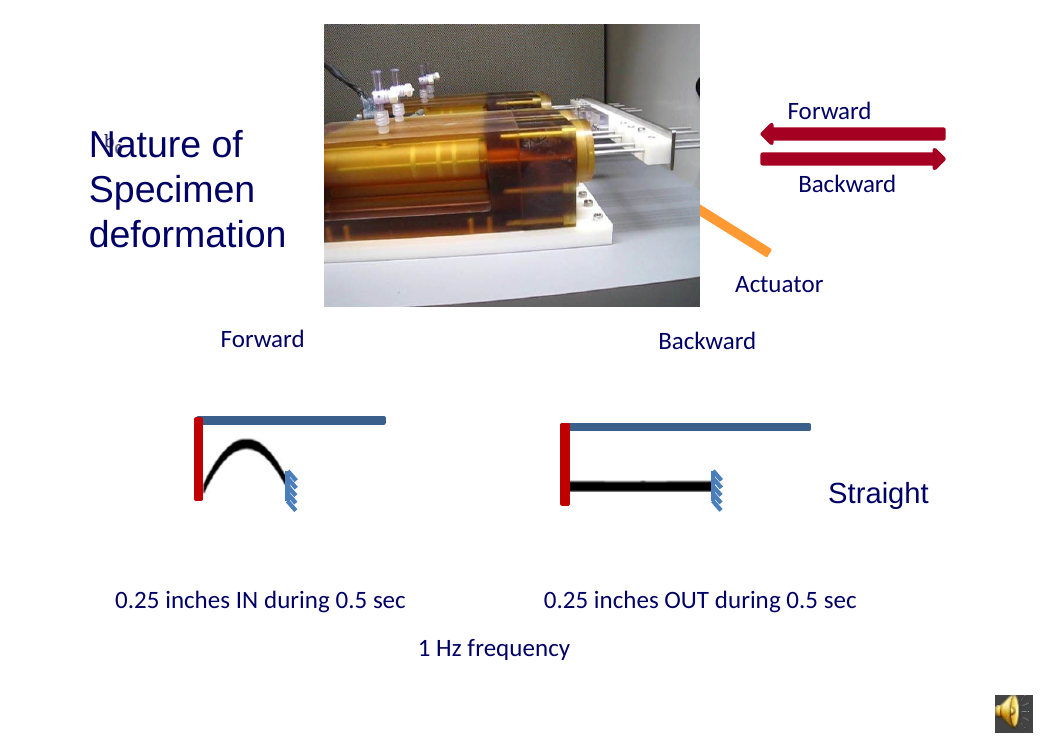

Forward
Nature of
Specimen deformation
Backward
Actuator
Forward
Backward
Straight
0.25 inches IN during 0.5 sec
0.25 inches OUT during 0.5 sec
1 Hz frequency

## Slide 4
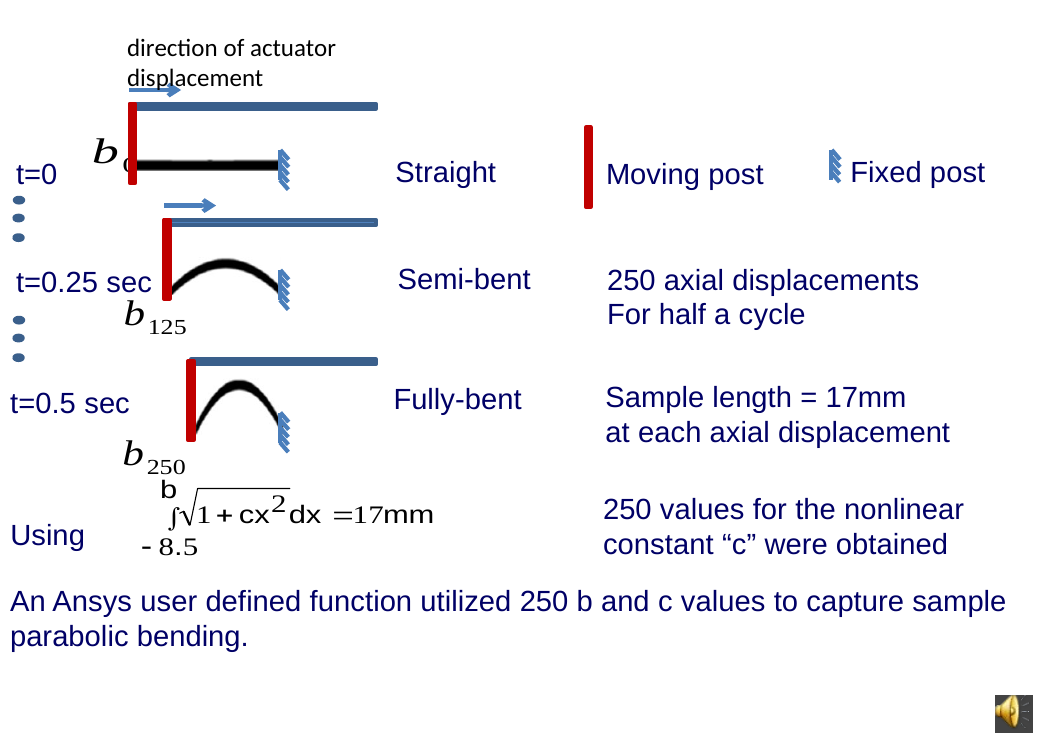

direction of actuator
displacement
Straight
Fixed post
Moving post
t=0
Semi-bent
250 axial displacements
For half a cycle
t=0.25 sec
Sample length = 17mm
at each axial displacement
Fully-bent
t=0.5 sec
250 values for the nonlinear constant “c” were obtained
Using
An Ansys user defined function utilized 250 b and c values to capture sample parabolic bending.

## Slide 5
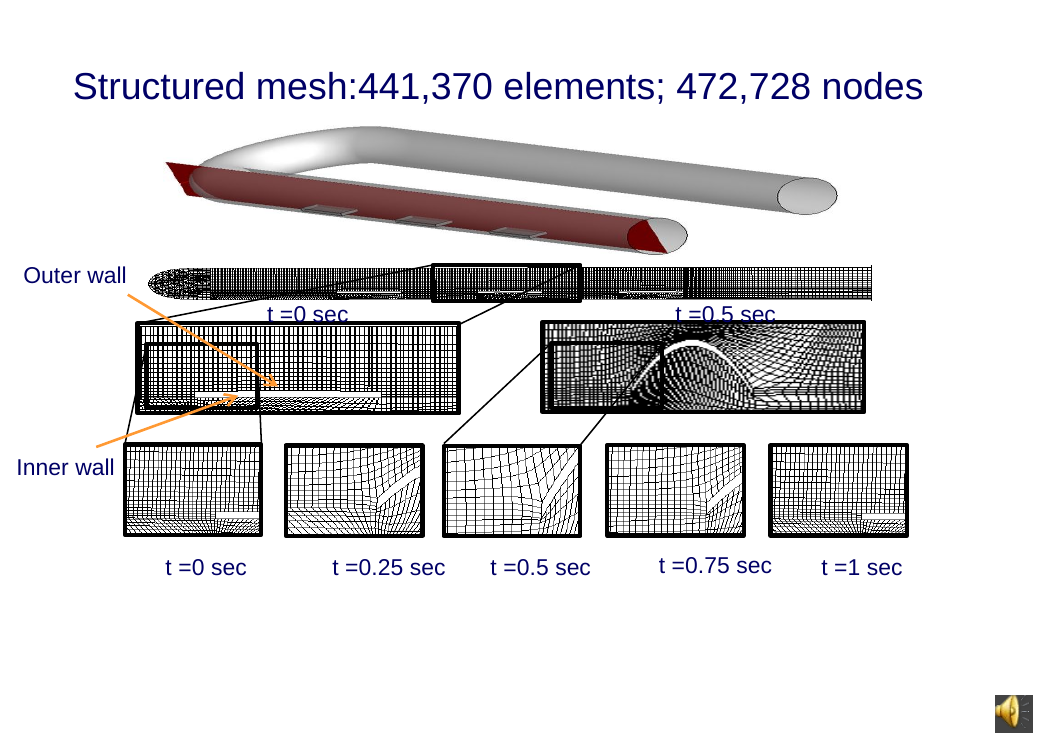

Structured mesh:441,370 elements; 472,728 nodes
t =0 sec
t =0.5 sec
t =0.75 sec
t =0.5 sec
t =0 sec
t =0.25 sec
t =1 sec
Outer wall
Inner wall

## Slide 6
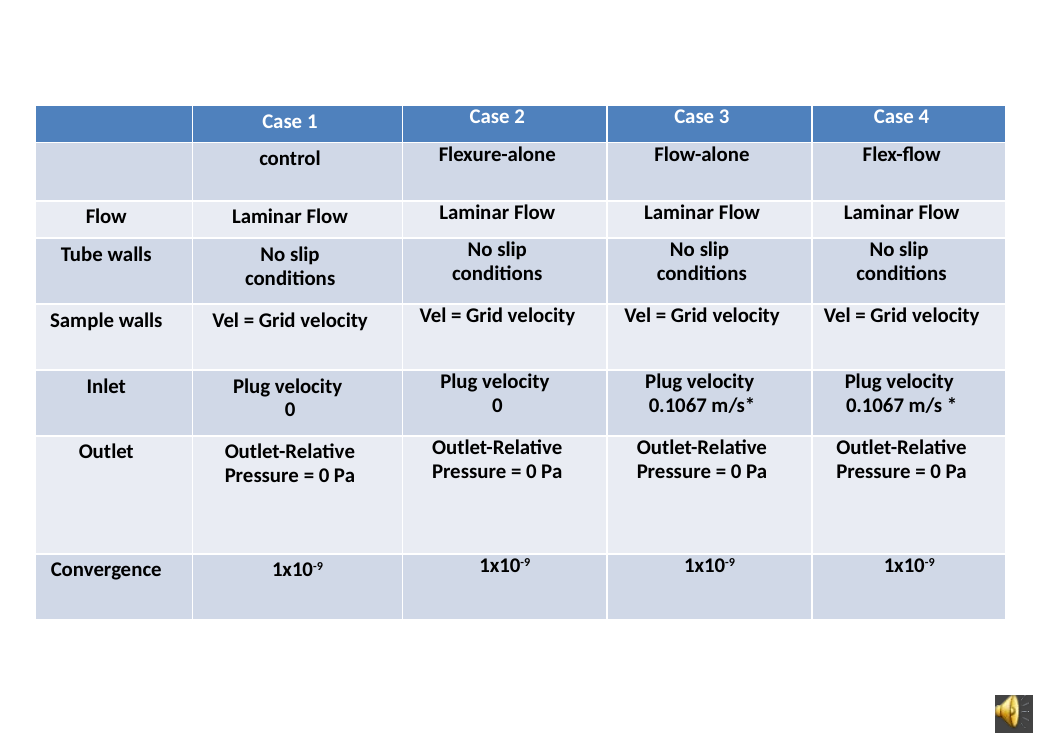

| | Case 1 | Case 2 | Case 3 | Case 4 |
| --- | --- | --- | --- | --- |
| | control | Flexure-alone | Flow-alone | Flex-flow |
| Flow | Laminar Flow | Laminar Flow | Laminar Flow | Laminar Flow |
| Tube walls | No slip conditions | No slip conditions | No slip conditions | No slip conditions |
| Sample walls | Vel = Grid velocity | Vel = Grid velocity | Vel = Grid velocity | Vel = Grid velocity |
| Inlet | Plug velocity 0 | Plug velocity 0 | Plug velocity 0.1067 m/s\* | Plug velocity 0.1067 m/s \* |
| Outlet | Outlet-Relative Pressure = 0 Pa | Outlet-Relative Pressure = 0 Pa | Outlet-Relative Pressure = 0 Pa | Outlet-Relative Pressure = 0 Pa |
| Convergence | 1x10^-9^ | 1x10^-9^ | 1x10^-9^ | 1x10^-9^ |
